# Review—Mathematical Formulations of Electrochemically Gas-Evolving Systems

**DOI:** 10.1149/2.0791813jes

**Published:** 2018-10-10

**Authors:** Amir Taqieddin, Michael R. Allshouse, Akram N. Alshawabkeh

**Affiliations:** 1 Department of Mechanical and Industrial Engineering, Northeastern University, Boston, Massachusetts 02115, USA; 2 Department of Civil and Environmental Engineering, Northeastern University, Boston, Massachusetts 02115, USA

## Abstract

Electrochemically gas-evolving systems are utilized in alkaline water electrolysis, hydrogen production, and many other applications. To design and optimize these systems, high-fidelity models must account for electron-transfer, chemical reactions, thermodynamics, electrode porosity, and hydrodynamics as well as the interconnectedness of these phenomena. Further complicating these models is the production and presence of bubbles. Bubble nucleation naturally occurs due to the chemical reactions and impacts the reaction rate. Modeling bubble growth requires an accurate accounting of interfacial mass transfer. When the bubble becomes large, detachment occurs and the system is modeled as a two-phase flow where the bubbles can then impact material transport in the bulk. In this paper, we review the governing mathematical models of the physicochemical life cycle of a bubble in an electrolytic medium from a multiscale, multiphysics viewpoint. For each phase of the bubble life cycle, the prevailing mathematical formulations are reviewed and compared with particular attention paid to physicochemical processes and the impact the bubble. Through the review of a broad range of models, we provide a compilation of the current state of bubble modeling in electrochemically gas-evolving systems.

As electrochemical cells and reactors continue to become more pervasive in applications such as fuel cells, desalination, and metal extraction, accurate numerical modeling of the different physics and scales of this problem requires careful consideration for system optimization. A particularly challenging feature of electrochemical systems is the generation of bubbles at the surface of the electrodes. These bubbles, a by-product of the chemical reactions taking place, can both hinder and aid the efficiency of the cell. While the rising of the produced gas bubbles promotes flow circulation in the cell, gas production has unwanted effects such as changing the conductivity of the bulk liquid and reducing the active area of the electrode surface.^1–3^ Efficient system design requires accurately modeling bubble generation, growth, and flow, which occurs at different length and time scales.^4^

Initially, gases are in a dissolved state inside the electrolyte’s liquid phase. However, due to the continuous production of these gases by the chemical reactions, the electrolytic medium becomes supersaturated and gas bubbles start forming at the electrode surface.^5^ Despite the importance of electrochemical gas-evolving systems and their frequent applications, further development and incorporation of several underlying physicochemical phenomena, such as bubble nucleation, electrical field and Marangoni stress effects on the bubble, and liquid-bubble momentum and mass exchanges, are required to accurately model the bubbles in the electrolyte.^6–9^ While all of these effects have been studied in isolation, their interactions and importance to other scales demands a multiscale, multi-physics framework to enable a comprehensive description of electrochemically gas-evolving system.

From its birth to eventual bursting, each phase of the life cycle of a bubble involves multiple phenomena at different scales. Understanding nucleation, the generation of the bubble, at the electrode surface requires the consideration of the electrolytic medium states and processes prior to the nucleation stage. Specifically, evaluation of the amount of the dissolved gases and the supersaturation condition are needed to determine nucleation rates. The next stage in the bubble’s evolution cycle is bubble growth at the electrode surface, which ends at the stage of bubble detachment. Accurately modeling the growth of a bubble is impacted by hydrodynamics and transport of species through the interface. After the bubble detaches from the electrode, the rising stage takes place where the bubble starts ascending through the electrolyte phase. Once the bubble has reached the electrolyte free surface, the bubble undergoes bursting, the final stage. During these five main stages, multiple, interconnected physics such as chemical reactions by the electrode current kinetics, generation of gases, species transport, and two-phase fluid interactions take place.

Extensive experimental work has been done to study the electrochemically produced gas-bubbles by means of employing Particle Image Velocimetry and Laser Doppler Anemometry techniques.^10–13^ These techniques, however, are limited to systems with either a single or small number of bubbles, or are restricted to investigating a single stage of the bubble life cycle, for example bubble growth dynamics, or bubble flow in two-phase system.^14–16^ Alternatively, numerically modeling the behavior of bubbles from a multiscale, multi-physics viewpoint provides an opportunity to investigate the contributions of multiple physics phenomena simultaneously throughout the life cycle of a large number of bubbles. Deriving models to predict the gas bubble evolution in electrochemical systems is challenging since the associated physics occur over vastly different time and spatial scales, ranging from nanometers, such as electronic transfer process that occurs at femtoseconds, to meters where hydrodynamics and species interaction effects take place on the order of seconds to hours. The nonlinearity in gas bubble behavior and the intrinsic physicochemical hydrodynamics further complicates models of the gas-evolving systems. For instance, understanding the bubble detachment from the electrode surface requires a careful consideration of the interfacial forces, sliding motion, coalescence, and jump off behavior of the bubble at the electrode surface.^17^ Predicting and simulating the electrochemically gas-evolving systems entails a successfully bridging technique that couples the multiscale physics within the system including bubble formation, growth, detachment, interfacial characteristics, ion transport, transport of species, electrolyte properties, and two phase dynamics. Although previous investigations have modeled the multiple scales of electrochemical systems, bubbles are not fully integrated into the models.^18–22^

Accurately accounting for bubbles in the design of electrochemical systems helps reduce the cell voltage drop and energy loss, increases the mass transfer rates, and protects the electrode surface.^3,23,24^ These consequences make incorporating gas bubble behavior into electrochemical systems crucial to modeling from a multiscale and multiphysics viewpoint. The multiphysics modeling using multiscale approach is a rapidly developing research area due to its ability in advancing the deep understanding of fundamental sciences. However to date, there has not been a comprehensive review of the existing isolated phenomena models and how they interact within the context of bubbles in electrochemically gas-evolving systems. In this paper, we present a review of the physicochemical aspects of the different phases of the bubble’s life cycle from a multiscale and multiphysics viewpoint.

We will provide a mathematical reference of multiscale formulations, to help researchers in modeling bubbles in electrochemically gas-evolving systems simulations. This starts with a discussion of multiscale coupling principles and introduction of the multiphysics models in the electrochemically gas-evolving systems. The next section compares nucleation theories at different scales and presents the associated mathematical framework. After bubble nucleation, we discuss the physical insight and the governing mathematical formulations of bubble growth. Bubble growth is due in part to the increased ion concentrations that are produced by the electrochemical kinetics near the electrodes. We present the three prevailing theories of electrochemical kinetics. Ultimately, bubble growth leads to detachment from the electrode surface, so we present detachment models where interfacial forces play a key role. Next, we present the dynamics of the bubble and transport of species through the interface as it rises in the two-phase flow section. Bubble bursting upon reaching the surface is not presented because it generally occurs outside the active domain of the electrochemical system. Finally, we provide an overview about the recent progress in the area of the gas-evolving systems and potential directions that could be pursued by implementing multiscale multi-physics modeling.

## Principles of Multiscale Physics

**Figure 1.**
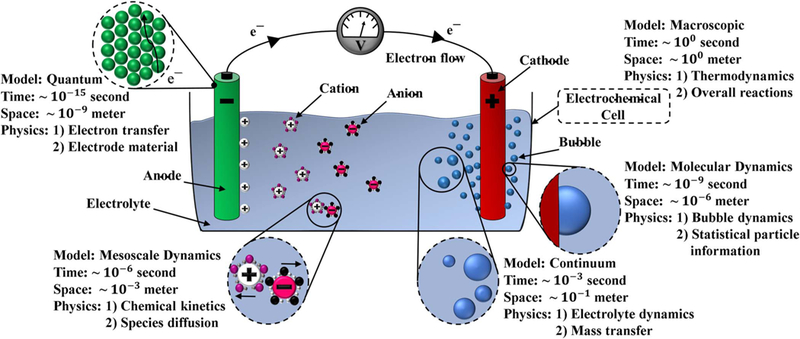
Schematic of relevant multiscale computational physics and the corresponding length and time scales pertinent to electrochemical gas-evolving systems.

Multiscale modeling aims to analytically or numerically link different physical models that span different degrees of simplifying assumptions and resolution to investigate a system. This type of modeling provides a comprehensive description of a multi-physics system because the impact of smaller scale effects can be accurately measured and communicated to the phenomena at larger scales, which reduces the computational cost of solving the physics entirely at small scales. Several models are needed to accommodate for the enormous breadth of spatial and time scales in electrochemical systems. For instance, the thickness of the electrical double layer, concentration diffusion layer, and viscous boundary layer are respectively 10^−9^, 10^−4^ and 10^−3^ meters.^13^ Figure 1 shows a sample multiscale computational models with the prevailing physics and corresponding temporal and spatial scales in the gas-evolving system.

The electrochemically gas-evolving system can be divided into five computational groups for simulation purposes based on the characteristic lengths and temporal scales. As shown in Figure 1, the computational models; quantum, molecular, mesoscale, continuum, and macroscopic models have spatial scales ranging from 10^−9^ to 10^0^ m and time scales from 10^−15^ to 10^0^ seconds. Selecting a physically consistent and computationally efficient approach to model each individual group and coupling the approaches of these groups together is a non-trivial process and is the focus of many investigations and precisely where many significant scientific advances can be made.

The finest relevant scale in electrochemical systems is the quantum model where the material properties and dependencies on atomistic structure are modeled. Quantum mechanics modeling describes the interactions between the electrode material and electron transfer. An increasingly powerful, accurate, and convenient computational tool for studying the electronic structure (i.e. electron transfer), the density functional theory (DFT) is one approach for modeling the quantum scale physics.^25^ Combining DFT with statistical mechanics is considered the most successful approach to modeling the electrochemically active materials.^20^ The combined approach reduces the computational cost and helps to overcome the computational limitations of the structure cell size.^26,27^ DFT formulation will be further presented in the context of bubble nucleation in which DFT is used to calculate the nucleation rates.

Electron transfer at the electrode initiates a number of reactive and non-reactive physicochemical processes in the electrochemical system that impact the molecular and mesoscale dynamics. The molecular dynamics approach can be used to simulate the solvation of ions in the electrolyte, their adsorption by the electrode surface, and stability of the double layer that forms above the electrode.^18^ One of the most common applications of molecular dynamic simulations of electro-chemical systems is quantifying the effective local molecular diffusivity and kinetics.^21^ At a slightly larger scale, mesoscale dynamics computational methods, such as discrete-time dynamics, dissipative particle dynamics, and population balance models, are used to efficiently bridge the gap between molecular dynamics and continuum scales.^28–33^ For instance, the mesoscopic scale can be used to model a microscopic quantum lattice-gas system implementing the lattice Boltzmann equations,^34^ or it can be used to describe a discretized continuum systems such as fluid particles.^31^

The fluid dynamics of the electrochemically gas-evolving system should be incorporated in both the mesoscopic and continuum models. The mesoscopic model is required to properly understand the interfacial bubble-surface or bubble-liquid interactions, and the continuum model represents the electrolyte flow using the Navier-Stokes equations. Continuum approximations are also used to model interfacial mass transfer. Finally, the macroscopic scale, which is the largest and based on the characteristics length of the electrochemical cell, is implemented to determine the mechanical and thermodynamic characteristics of the cell.

**Figure 2.**
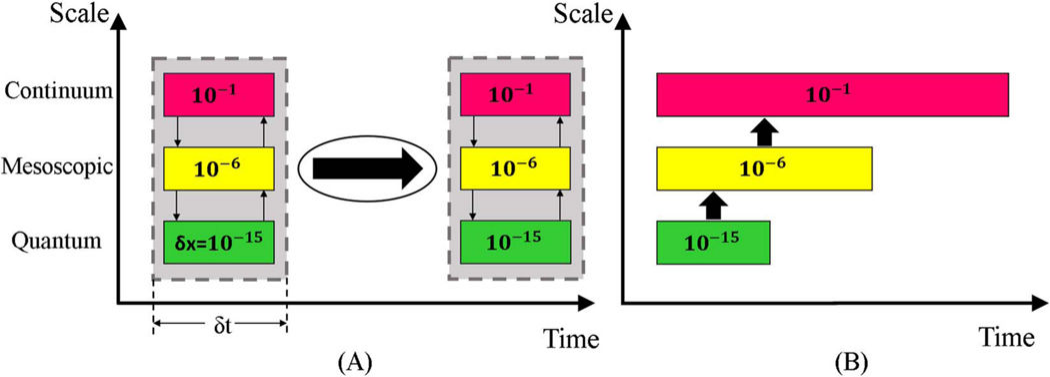
Schematic of multiscale coupling strategies. (A) Concurrent coupling method requires solving all models at the same time step in a parallel mode then moving to the next time step. (B) Sequential coupling method requires solving models over different time lengths sequentially then passing parameters from one model to another.

Bridging the various models at different scales is the main goal of multiscale modeling. The two main strategies to couple the different scale physics together into a single coherent system are the concurrent and sequential coupling methods.^18,35^ Figure 2 shows the schematic approach of the two coupling strategies. The concurrent or parallel coupling method requires construction of partitions of the physical domain, each partition corresponds to a single physical domain at a given spatial and time scales. Information transfer between these regions occurs continuously between each partition of the domain. For example, the macroscopic scale uses the information from the micro scale as the computation proceeds using the “on-the-fly” concept.^36^ The concurrent approach links the different partitions via a set of boundary conditions. Defining the interfaces between these partitions and the corresponding boundary conditions with physically consistent models is a challenging task. Compounding this challenge is the fact that treating interfaces as discontinuities is complicated by the boundaries moving with time. Finally, the time marching of the concurrent model is set based on the smallest time step of the finest model since all partitions are solved together at the same time. This can substantially increase computational costs for systems that need to run for long time. Several techniques have been developed to simulate the multiscale systems concurrently, such as multiscale field theory, handshaking domain, heterogeneous multiscale, and quasi-continuum methods.^37^

An alternative to the concurrent technique is the sequential or serial coupling technique that is based on simulating one scale at a time and transferring information from the small-scale models to the larger scale models.^38^ Serial stitching is thus known as a parameter passing approach in which the variables are parametrized based on results at the micro scale computations and then passed to the next larger models. Passing the unknown parameters between the single physical models requires a reliable strategy to fit and transfer these parameters.^38^ Parameter passing requires the implementation of phenomenological theories to derive the key parameters of the large-scale models from the smaller models. These derivations are based on simplifications and local approximations that limits this technique to only a few key parameters.^36^ In fact, the computational cost of fitting the full constitutive relations (parameters) is too expensive to gather highly accurate information from the smaller scales to the larger scale models, so power law relationships are often used where coefficients are determined from the smaller scale models.

Both the sequential and concurrent techniques have their own strengths and weaknesses. Of the two methods, the sequential technique is generally considered to be more efficient computationally and the more well-established method despite the challenge of parameter passing.^38^ The concurrent coupling approach is a more intricate and computationally demanding method due to the limitation of the time step and complexity of domain partitioning of the system. On the other hand, the concurrent technique has more general applicability compared to the sequential approach since it does not require any phenomenological models.^38^ To enhance the coupling effectiveness of multiscale simulations a hybrid coupling scheme could be implemented, using both concurrent and sequential strategies. The purpose of the hybrid approach is to benefit from combining elements from both the parallel and serial models by reducing the associated restrictions in partition of domains and lower the number of the needed phenomenological models.^38^ While the hybrid approach is very promising, it requires further development before being applicable to electrochemical systems with bubbles. The bubble models we present are applicable to both the sequential and concurrent approaches.

## Bubble Nucleation

**Figure 3.**
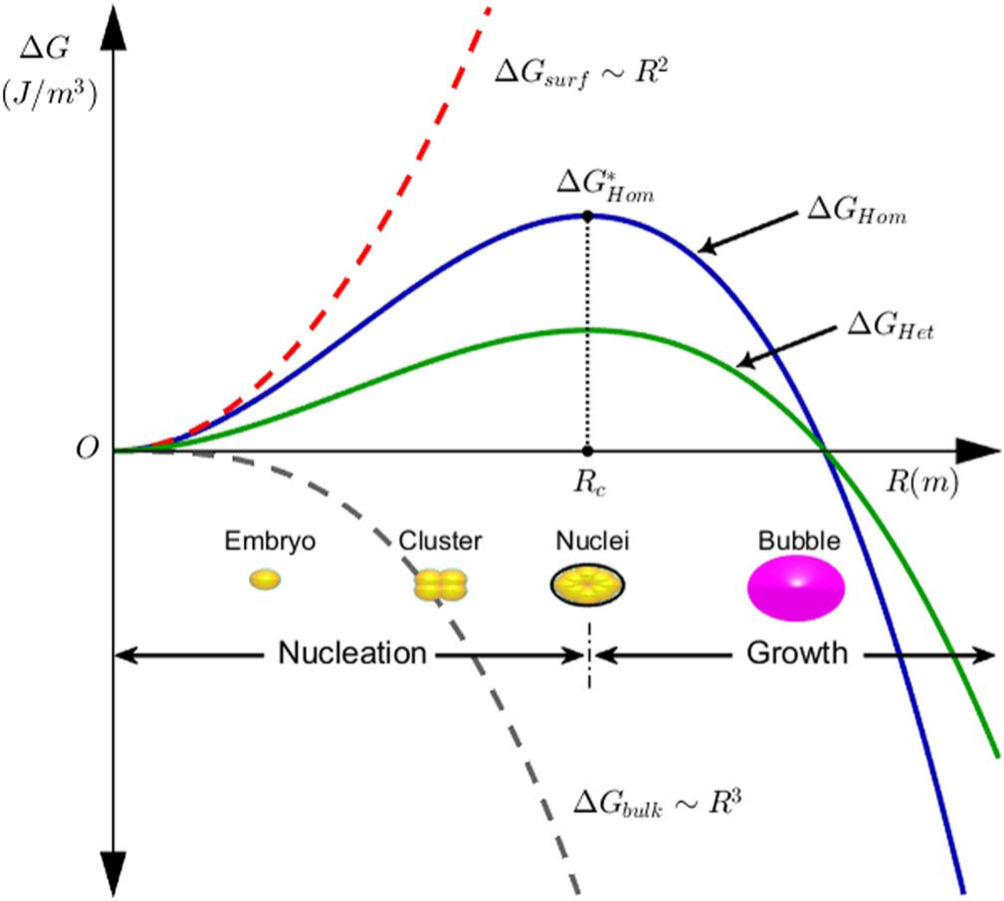
Gibbs free energy change during bubble nucleation. The surface free energy Δ*G*_*sur f*_ (dashed red line), bulk free energy Δ*G*_*bulk*_ (dashed black line), homogeneous nucleation free energy Δ*G*_*Hom*_ (blue line), and the heterogeneous nucleation free energy Δ*G*_*Het*_ (green line) are presented as a function of the radius *R*. The nucleation stages are also sketched for reference. The embryo and cluster states are reversible and the nuclei state is irreversible. Beyond the nuclei phase the bubble starts growing.

While the life cycle of bubbles begins at the nucleation stage, the precursor to this is the generation of dissolved gas by electrochemical reactions. The nucleation process is defined as the physicochemical transformation of particles initially in the metastable phase of high concentrations of dissolved gas in the electrolyte to the more stable phase of a gas bubble. Nucleation occurs when the supersaturation condition is satisfied at which point the concentration of the dissolved gases in the liquid is high enough to initiate the nucleation. Bubble nucleation begins by a molecular formation of particles, known as embryos, from the combination of the solute molecules. These embryos are a dense collection of the dissolved gas particles in a metastable state meaning they can spontaneously dissolve or can become bubbles. Consequently, a close examination of the transformation of these groups of molecules is crucial to understand the nucleation phenomena. The unstable intermediate formation of embryos requires a sufficient amount of energy to overcome the activation barrier of the solute phase transformation. Embryos may grow and assemble to form a cluster, on the order of nanometers, or decay and dissolve again in the liquid phase. Similarly, the cluster stage is reversible and it can decay. Once the cluster gains the required activation energy an irreversible phase change occurs with a critical size, which is known as the nuclei, as shown in Figure 3. The nuclei is a stable state and will not spontaneously revert back to the dissolved gas state. Beyond the critical point, the nuclei grows forming a macroscopic single bubble.

Bubble nucleation can be classified either as homogeneous or heterogeneous nucleations. Homogeneous nucleation happens in a medium of solute and solvent, without the presence of any surfaces or impurities.^39^ For example, bubbles can nucleate spontaneously inside a bulk liquid during boiling. Heterogeneous nucleation occurs at the interfaces with the presence of substrates, which can be any foreign body such as the electrode or a preexisting bubble. Normally, substrates lower the required interfacial free energy required for nucleation.^40^ The substrate can impact the nucleation rate in two ways. First, because the surface is not perfectly smooth and not perfectly wetted by the electrolyte, nucleation sites will naturally form where small pockets of gas are trapped to the surface. Second, by providing a surface on which to attach there is a lower surface area exposed to the electrolyte. This reduces the free energy necessary to form the surface of the bubble. Properties of the substrate can also impact the nucleation rate. For instance, coating of the nucleation surface changes the substrate topography and chemistry resulting in either higher or lower wetting contact angle between the fluid and the substrate, which will impact the nucleation rate. Also, nucleation rates can change when a surface is heated because the surface tension decreases as the temperature increases.^41^

Classical nucleation theory (CNT) is the standard and most widely used theory to model bubble nucleation. CNT predicts the nucleation rate by describing the activation barrier to producing a nuclei in terms of a combined thermodynamic and kinetic activation potential, which is the Gibbs free energy, Δ*G* (J). The free energy of homogeneous nucleation, Δ*G*_*Hom*_, is described using CNT as the sum of two components: a surface free energy term, Δ*G*_*surf*_, which makes a positive contribution and a bulk free energy term, Δ*G*_*bulk*_ that makes a negative contribution. Accordingly, Δ*G*_*Hom*_ can be given as a function of the bubble’s radius, *R* (m), as follows:

[1] $$\Delta G_{Hom}=4\pi \sigma R^{2}-\left(4\pi /3\right)\Delta g_{\upsilon }R^{3},$$

where σ (N m^−1^) represents the interfacial surface tension at the gas/electrolyte interface and Δ*g*_*v*_ (J m^−3^) denotes the energy density difference between the dissolved and gaseous states of the molecules in the bubble.^42^ The first term in the right-hand side of Eq. 1 represents Δ*G*_*surf*_ and the second term is Δ*G*_*bulk*_. CNT can account for the free energy of heterogeneous nucleation, Δ*G*_*Het*_, by introducing a geometric function, *f* (θ), to consider the energy reduction due to the presence of the substrate. The behavior of *f* (θ) depends on the wetting angel, θ, at the substrate interface. The heterogeneous nucleation free energy is given as $\Delta G_{Het}=f\left(\theta \right)\Delta G_{Hom}$.

Bubble shrinkage or growth depends on the value of Δ*G*. As shown in Figure 3, the bubble grows only once it reaches a critical radii, *R*_*c*_, at which point the provided free energy reaches its maximum, ${\Delta G}_{\mathrm{Hom}}^{*}$. Prior to reaching *R*_*c*_, the bubble is defined as sub-nuclei and its natural tendency is to shrink. In electrolytic mediums, bubble nucleation is driven by thermodynamic supersaturation, ζ, which is the ratio of the dissolved gases concentration adjacent to the electrode surface, *c*_*e*_ (mol m^−3^), and the equilibrium, far field dissolved gases concentration in the bulk liquid phase, *c*_∞_ In general, nucleation requires ζ to exceeds 100.^43^ In cases where the bulk concentration is significantly depleted due to a large number of bubbles generated, a higher activation energy is required for nucleation. However, the threshold of ζ required for nucleation shows only small variations.^43^

There are many contributing factors that impact the modeling of nucleation at the electrode surface requires.^44^ Electrode adsorption of molecules can impact the nucleation rate at the electrode surface. Cations and anions change Δ*G* and influence the discharge kinetics of ions, which in turn impacts the chemical reaction process. This can impact the production of the dissolved gas. Additionally, these processes can influence the surface tension, which also modifies the nucleation rate. Taking into account all these factors, the rate at which the clusters turn into nuclei stage in units of nuclei per a unit volume per a unit time, *J*_*o*_(nuclei m−3 s−1), can be given in terms of the maximum energy of heterogeneous nucleation, ${\Delta G}_{\mathrm{Het}}^{*}$, at the substrate as:^44^

[2] $$J_{o}=DC\exp\left(-\frac{\Delta G_{Het}^{*}}{k_{B}T}\right),$$

where *D* (mol m^−3^ s^−1^) denotes the flow of molecules that are added to the forming nuclei, *C* (nuclei mol^−1^) is the concentration of molecules (nuclei consisting number of molecules), *k*_*B*_ = 1.38 × 10^−32^ (J K^−1^) is Boltzmann’s constant, and *T* (K) is the electrolytes medium temperature. In this case, both ${\Delta G}_{\mathrm{Het}}^{*}$ and *R*_*c*_ can be expressed in terms of the electrode overpotential, η (V), as:

[3] $$\Delta G_{Het}^{*}=f\left(\text{θ}\right)\frac{16\pi \sigma^{3}V^{2}}{3{\left(zF\text{η}\right)}^{2}},$$

[4] $$R_{c}=\frac{2\sigma V}{zF\text{η}},$$

where *V* (m^3^ mol^−1^) is the specific volume of the nucleating bubble, *z* is the transfered ion charge, and *F* = 96485 (C mol^−1^) represents Faraday’s constant. For the spherical cap model of bubble nucleation, which is the most common nucleation model, the geometric factor is $f\left(\text{θ}\right)=1/4\left(2+cos\text{θ}\right){\left(1-cos\text{θ}\right)}^{2}$.^39^ The overpotential is defined as the difference between the electrode potential and the equilibrium potential of the chemical reaction. In Eqs. 3 and 4, the critical heterogeneous free energy and radius are defined in terms of η based on the thermodynamic equilibrium and ions activity.^45^

Despite the fact that CNT serves as a benchmark for determining Δ*G*, the computed nucleation rates by CNT underestimate the experimentally measured nucleation rate.^46^ CNT makes several assumptions to compute the nucleation rates that may contribute to the error in predicting the nucleation. These assumptions include:

1. The nucleation rates in CNT are determined from a macroscopic viewpoint of the surface tension and it neglects the curvature corrections to Δ*G*_*surf*_.^47^ Electrodes may have cylindrical, pin or mesh shape. In these cases, modeling of bubble nucleation at non-planar interfaces requires adding curvature corrections to calculate the nonuniform surface tension.
2. CNT computes the nucleation rates based only on the nucleus size and ignoring the effect of local fluctuation and depletion of the solution concentration.^39^ For instance, consumption of molecular concentration changes the free energy of nucleation and may effect the supersaturation limits that drives the bubble nucleation.
3. The sub-nuclei states are assumed to take place under thermodynamic equilibrium and bubble formation is assumed to take place at steady state. However, the supersaturation changes during nucleation result in unsteady nucleation rates that lead to varying the nucleation activation energy limits.^48^
4. In electrochemical systems, the ionic strength of the electrolytic medium plays a significant role in the bubble nucleation. The ionic species adsorption at the bulk solution/bubble interface changes the interfacial surface tension values, which impacts the nucleation rate.^6^

In contrast to the macroscopic view of CNT, the density functional theory (DFT), a computational quantum scale model, uses a molecular scale approach to calculate the nucleation rates.^49–51^ In the DFT approach, the nucleating medium is assumed to have inhomogeneous molecular structure where the metastable phase is described by a spatially varying density function. In this molecular-based approach, the potential of short-range interactions, ϑ(***r***) is

[5] $$\vartheta \left(r\right)=\vartheta_{rep}\left(r\right)+\vartheta_{att}\left(r\right)$$

where $\vartheta_{rep}\left(r\right)$ is a leading order repulsive potential, $\vartheta_{att}\left(r\right)$ (J m^−3^) is a perturbation attraction potential, and ***r*** (m) is a position in the fluid. The repulsive component represents the structure of the fluid and the attractive potential models the local density of the fluid. The intrinsic free energy of the system, ψ[ϕ(***r***)] (J), can be expressed as a function of the probability density profile, ϕ(***r***) (m^−3^) in the form:

[6] $$\begin{array}{l} \psi \left[\text{ϕ}\left(r\right)\right]\\ \;=\int f_{h}\left[\text{ϕ}\left(r\right)\right]dr\;+\frac{1}{2}\int \int \text{ϕ}\left(r\right)\text{ϕ}\left(r^{\prime }\right)\vartheta_{att}\left(\left|r-r^{\prime }\right|\right)drdr^{\prime }\\ \;\;\;+O\left(\vartheta_{att}^{2}\right), \end{array}$$

where $f_{h}\left[\text{ϕ}\left(r\right)\right]$ (J m^−3^) is the density of Helmholtz free energy of a reference system, and $O\left(\vartheta_{att}^{2}\right)$ denotes the order of error due to expanding the perturbation potential into a first order approximation. The grand free potential energy, $\Omega_{DFT}$, can be written in terms of chemical potential, χ (J), as following:

[7] $$\Omega_{DFT}\left[\text{ϕ}\left(r\right)\right]=\text{ψ}\left[\text{ϕ}\left(r\right)\right]-\text{χ}\int \text{ϕ}\left(r\right)dr$$

where the equilibrium density profile is determined as the saddle point of $\Omega_{DFT}$ such that $\partial \Omega_{DFT}/\partial \text{ϕ}=0.$ Even though CNT and DFT differ in the calculation of the free energy, both share the same pre-exponential factor in Eq. 2. Also, the predicted nucleation rate by DFT is the same calculated in the vicinity of thermodynamic equilibrium of phase coexistence. However, DFT shows more dependence on the temperature and higher accuracy over CNT away from equilibrium.^52,53^ DFT represents a promising technique to determine the nucleation rate, but this technique has yet to be applied to bubble nucleation in electrochemical systems.

The nucleation stage is associated with other coincident physics. Evaluation and investigation of bubble nucleation requires accurate computing of species concentration to determine the concentration ratio condition, ζ, and to calculate the nucleation rate in terms of *C* in Eq. 2. Furthermore, the electrochemical cell property η and the chemical reaction ion transfer parameter *z* are needed as well. Determination of these quantities will be discussed as part of electrochemical kinetics impact on bubble growth.

## Bubble Growth

Bubble growth in an electrolytic medium involves material transfer from the liquid phase to the gas bubble phase through the interface. The rate of bubble growth at the electrode surface depends on the concentration ratio, which drives the interfacial mass transfer via diffusion through the bubble’s interface per Fick’s law at the macroscopic scale.^54^ Interfacial mass transfer flux, $\dot{m}\prime \prime$, can be expressed as follows:

[8] $$\dot{m}\prime \prime =k_{m}\left(c_{\infty }-c_{o}\right),$$

[9] $$c_{o}=k_{H}p_{o},$$

where *c*_*o*_ is the concentration at the bubble interface, *k*_*m*_ (m s^−1^) is the interfacial mass transfer constant, *k*_*H*_ (mol m^−3^ Pa^−1^) is Henry’s constant and *p*_*o*_ (Pa) is the pressure inside the bubble. Eq. 9 represents Henry’s law which is an equilibrium law that states the amount of dissolved gases, in a given type and volume of a liquid, is directly proportional to the partial pressure of that gas by the proportional factor *k*_*H*_. The value of *k*_*H*_, which is determined experimentally, depends on the solubility of the gases which depends on the system temperature; when the solubility decreases as the temperature increases, *k*_*H*_ decreases.

Several mass transfer processes take place at the surface of the gas-evolving electrodes including mass transfer from the liquid solution to the electrode surface, adsorption of the ionic gas reactant at the active sites on the electrode surface, diffusion of the gaseous products toward the liquid bulk, and mass transfer of the dissolved molecular gas product into the surface of the growing bubble.^55^ These processes determine the amount of molecular adsorption by the bubble interface which controls the bubble growth rate. The total amount of the generated gases at the electrode surface that will be either dissolved or eventually form the bubble, *j* (mol s^−1^), is given by Faraday’s law:

[10] $$j=\frac{I}{zF},$$

where *I* (A) is the applied current at the active electrode. The gas produced by the electrode can then either be absorbed into the bubbles resulting in growth or remain in the electrolyte and increase the local supersaturation ratio. While there is some variation in the dissolved gas concentration, it is frequently assumed that the dissolved gas is uniformly distributed inside the electrolytic liquid. This removes the need to consider spatial derivatives when deriving the time rate of change of the concentration of dissolved gas in the electrolyte, *d*(*cs*)/*dt* (mol m^−3^ s^−1^), which is modeled as:^55^

[11] $$V_{\ell }\frac{d\left(c_{s}\right)}{dt}=\frac{1}{zF}-A_{b}\dot{m}\prime \prime ,$$

where *V*_ℓ_(m^3^) is the volume of the liquid solution and *A_b_* (m^2^) is the bubble’s surface area. If the interfacial mass transfer is limited because the diffusivity of the gas through the interface is small resulting in reduced bubble growth and a build up of dissolved gas in the electrolytic bulk. If *k_m_* is relatively large, then the amount of transferred gases through the bubble surface is significant and the growth rate will be fast.^55^

The presence of the bubbles attached to the electrode surface causes portions of the surface to be inactive, which prevents the electrochemical reaction from proceeding at these inactive spots.^8^ Therefore, the fraction of the surface covered by bubbles, Θ, will play a large roll in the amount of dissolved gas produced, which in turn impacts nucleation and bubble growth rates. In important quantity needed for estimating bubble growth is the fraction of the total gas produced that is eventually transfered into the attached bubbles, *f*_*g*_.^56^ Based on a theoretical approximation, *f*_*g*_ can be expressed as:^8^

[12] $$f_{g}=0.55\Theta^{0.1}+0.45\Theta^{8}.$$

This implies that when there are no bubbles present, Θ = 0, all produced gases from the reactions are dissolved in the bulk meaning that *f*_*g*_ = 0. In contrast, as the electrode becomes completely covered, Θ→1, the little gas that is produced is prevented from reaching the bulk and is absorbed by the bubbles, *f*_*g*_ → 1.

**Figure 4.**
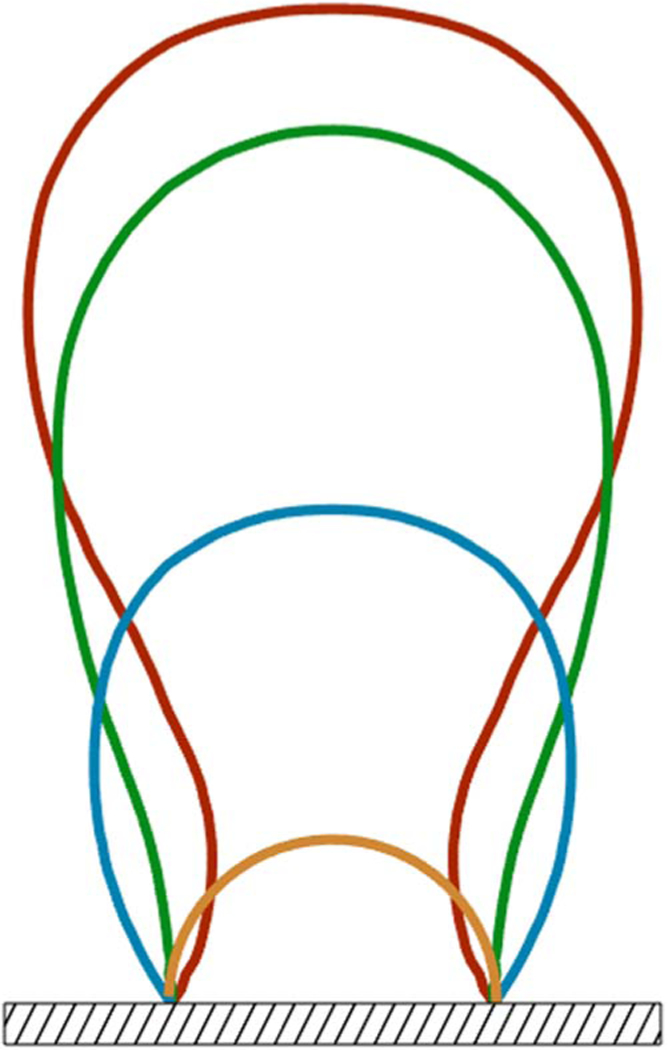
A sketch of the stages of bubble growth before detachment from a substrate: nuclei (orange line), under critical growth (blue line), critical growth (green line), and necking (red line).

Bubble growth evolves in several phases. Starting from an initial radius during the nucleation stage and ending at the necking stage prior to the detachment from the electrode surface as shown in Figure 4. However, in between these two phases, the bubble growth behavior is given in the general form *R*(*t* ) = *c*_*R*_*t^s^*, where the value of the fitting coefficient *c*_*R*_ and *s* are determined by the physicochemical conditions that control the bubble growth.^55,57,58^ This simplified model of the radius growth is not dependent on the surface shape. If the bubble grows rapidly then the growth kinetics are driven by the inertial effects and the growth is known as inertia-dominant growth, in which *s* equals 1.^57,58^ The inertia-dominant growth exists when a vapor bubble grows in a superheated liquid domain under low pressures.^59^ During slow bubble growth, the growth behavior is known as diffusion-controlled because gas diffusion across the bubble interface drives the bubble growth and in this case *s* will be on the order of 1/3 or 1/2.^2,57,58^ The criteria for selecting the value of *s* is related to the value of the applied current at the electrode. Generally, the rate of bubble growth, $\dot{R}$, for diffusion-controlled growth in supersaturated solution can be expressed as:

[13] $$\dot{R}=\frac{MW\left(c_{\infty }-c_{o}\right)}{{\text{ρ}}_{\ell }}\sqrt{\frac{D_{\ell }}{\pi t}}\left(1+\sqrt{\frac{\pi D_{\ell }t}{R}}\right),$$

where *M W* (kg mol^−1^) is the molecular weight of the gas, *D_ℓ_* (m^2^ s^−1^) is the mass diffusivity coefficient of gas in the electrolyte.^57^ Given this scaling, relationship, it is possible to show that Eq. 13 can be simplified as *R* = *c_R_t*^0.5^. ^60^ The bubble growth behavior of *R* = *c_R_t*^0.5^ in electrochemical systems has been observed experimentally.^61^

While the bubble growth in electrochemical systems is modeled well by the diffusion-controlled formula of *R* = *c_R_t*^0.5^, at high current densities the bubble growth is more accurately modeled by *R* ∝ *t*^1/3^.^62^ At these high current densities, the chemical reactions at the electrode surface are fast enough to produce large amounts of gas that diffuses rapidly in the liquid solution. In this case, the chemical reaction rate producing the gas becomes the rate-limiting factor that controls the bubble growth.^58^ It should be noted that the reported bubble growth kinetics rates are restricted for a moderate current (<100 A cm^−2^) density and absence of bubble coalescence.^5^ Bubble growth behavior is attributed by the current density namely the electrode kinetics.

### Electrochemical kinetics.—

As given in Eq. 11, both the current and the electric potential determine the gas production rate at the electrode. In fact, the electrochemical oxidization and reduction reaction rates are controlled by the the electrode kinetics.^63^ The kinetics of the chemical reactions are realized in the electron transfer process within the electric double layer between the reduced and oxidized species.^64^ A charge-electron transfer reaction involves either gaining (losing) electrons by the electrode from (to) the active reacting species. Understanding the behavior of the electron transfer relies on solid-state physics to study the electronic and geometric effects on the electrocatalysis, material science to investigate the electrode composite and characteristics, and thermodynamics to study the reaction activation process.^65^

Several modeling theories have been developed to model the electrode kinetics ranging from the quantum to macroscopic mechanical scales.^64^ The Butler-Volmer (BV) kinetics model is the standard model of electron transport across the electrode-electrolyte interface is described from the macro-scale perspective.^66–68^ The BV approach relates the net current density *i* (A/m^−2^), and the electrode overpotential η (V), which is the difference between the electrode potential and the equilibrium potential of the reactions.^69^ The net current density is

[14] $$i=i_{0}\left[\text{exp}\left(\frac{{\text{β}}_{a}nF}{\bar{R}T}\text{η}\right)-\text{exp}\left(\frac{-{\text{β}}_{c}nF}{\bar{R}T}\text{η}\right)\right],$$

where *i*_0_ (A m^−2^) is the exchange current density, *n* is the number of transfered electrons per reaction, $\bar{R}$(J K^−1^ mol^−1^) is the ideal gas constant, and β_*a*_ and β_*c*_ are respectively the oxidation and reduction electron transfer coefficients, which indicate the favored reaction direction (forward or backward) based on the applied electric potential. The formulation of the net current density represents the total sum of the cathodic and anodic current, which act in opposite directions. To accurately account for the bubbles covering the electrode, it is impotant to multiply *i* by (1−Θ), where Θ denotes the inactive fraction of the electrode.^70^

The most common type of chemical reaction induced by the electrode is the single step reaction. For a single step reaction (*n* = 1), β_*a*_*+*β_*c*_ = 1.^71^ In this case, the electron transfer coefficients can be written as β_*a*_ = 1 − β and β_*c*_ = β, where β is known as the symmetry factor. Typically the electron transfer at both the anode and cathode are symmetric, so β = 0.5.^72^ In this case, the BV model can be simplified for a single step reaction as

[15] $$i=2i_{0}\sinh\left(\frac{F}{2\bar{R}T}\text{η}\right).$$

For large magnitude electrode overpotentials, the value of one of the exponential terms becomes negligible in Eq. 14. Then the overall current density can be approximated using the Tafel approach for a single step reaction as follows:

[16] $$i=\{\begin{array}{c} i_{0}\text{exp}\left(\frac{\left(1-\text{β}\right)F}{\bar{R}T}\text{η}\right)\;\;\left(1-\text{β}\right)F\text{η}\gg \bar{R}T,\\ -i_{0}\text{exp}\left(\frac{-\text{β}F}{\bar{R}T}\text{η}\right)\;\;\text{β}F\text{η}\ll -\bar{R}T.\;\; \end{array}$$

The Tafel approximation is valid when the contribution of the anodic or cathodic current is less than 1% of the net current. Another limiting case for the BV model occurs at low overponetials when the electrode kinetics of Eq. 14 can be linearized. For a single step reaction, the linearized formula is:

[17] $$i=i_{0}\frac{F}{\bar{R}T}\text{η}.$$

The electrochemical kinetics are directly proportional to the value of *i*_0_. Consequently, having fast kinetics requires imposing *i*_0_ high as possible.

The exchange current is defined as the current that flows at equilibrium. At equilibrium the net current is zero; however, the faradaic activity does not stop. At equilibrium, both the anodic and cathodic have the same magnitude, equal to the exchange current, but the opposite signs. A high current exchange density corresponds to fast reaction kinetics. The value of *i*_0_ depends on the nature and temperature of the electrode surface and the composition of the electrode-electrolyte interface solution.^73^ The exchange current density can be expressed as follows:

[18] $$i_{0}=nFk_{a}^{1-\text{β}}k_{c}^{\text{β}}c_{a}^{1-\text{β}}c_{c}^{\text{β}},$$

where *k*_*a*_ and *k*_*c*_ (m s^−1^) are the rate constants of the anodic and cathodic reactions, respectively, and *c*_*a*_ and *c*_*c*_ (mol m^−3^) are the concentration of the anodic and cathodic reactants. Eq. 18 is typical only valid for dilute solutions. For concentrated solutions, the configurational entropy and enthalpy, electrostatic correlations, and coherency strain all impact the exchange current in nonlinear ways.^74^ The exchange current density for concentrated solutions is modeled based on the activities of reacting species and a transition activity coefficient, γ_‡_, by Bazant (2013) as follows:^74^

[19] $$i_{0}=\frac{nek_{0}a_{a}^{\text{β}}{\left(a_{c}a_{e}^{n}\right)}^{1-\text{β}}}{\gamma_{‡}},$$

where *e* (C) is the elementary charge, *k*_*o*_ is the reaction rate constant, *a*_*a*_ and *a*_*c*_ are respectively the activity coefficients of the anodic and cathodic reactants, and *a*_*e*_ is the activity of the electrons. The order of the reaction determines the scale and the units of Eq. 19 parameters. Eq. 19 is derived by considering thermally activated transitions in an excess chemical potential energy surface with an electric field across the reaction coordinate contributing to transitions in state.^75^

The BV model and its limiting cases provide a good model for the electrochemical kinetics under normal conditions from a macroscopic viewpoint.^76^ Even though the BV model can be easily implemented, it does not yield substantial insight into the influence of the molecular nature of the electrode, the medium (solvent, supporting electrolyte), or the specific electroactive species in the electron transfer kinetics, which all depend on the charge transfer process.^63,64^ For large overpotentials, the resulting current should be saturated, but the BV model does not capture this. To more accurately model large overpotential systems, the molecular nature of the reaction needs to be accounted for which models electron transfer in electrode kinetics computing. Furthermore, the macroscale BV model fails to account for reaction dependency on the electrode material because of the double layer effects, structure of Helmholtz layer, energy distribution of the electronic states in the material which occurs. Also, the BV model does not consider interactions between the reactants and products that involve changes in the free energy of the electrolyte, such as the ion bonding and electrostatic interactions. Thus, a detailed model at microscopic level is required to understand the electrochemical kinetics accurately and overcome the limitation of BV model.

To investigate the electron transfer from a microscopic viewpoint, Marcus theory has been applied to study the electrode kinetics of electron transfer reactions at the molecular scales.^77,78^ This model provides more physical insight by describing the electronic transitions in terms of the electron reactions between the molecules by relating the activation barrier of the electron transfer to the free energy, λ (eV), required to reorganize the local atomic configuration without charge transfer.^64^ Originally, Marcus theory was developed for electron transfer kinetics between the same phase, and later the theory was modified to include the multi-phase transfer.^79,80^ For a high concentration solute, the Marcus model gives:^74^

[20] $$i=i_{0}\text{exp}\left(\frac{{\left(ne\text{η}\right)}^{2}}{4k_{B}T\lambda }\right)\left(\text{exp}\left(\frac{-\text{β}ne\text{η}}{k_{B}T}\right)-\text{exp}\left(\frac{(1-\text{β)}ne\text{η}}{k_{B}T}\right)\right).$$

Marcus model (Eq. 20) shows the more a more complex dependence on overpotential compared to BV model. The modified symmetry factor, β, is^74^

[21] $$\text{β=}\frac{1}{2}\left(1-\frac{k_{B}T}{\lambda }\right),$$

which is related to the reorganization energy. For thermally stable case, λ ≫ *k_B_T*, β = 1/2, which matches the BV model.

Further developments of the Marcus theory have led to the more accurate Marcus-Hush-Chidesy (MHC) kinetics model.^81,82^ The oxidization (ox) and redox (red) chemical kinetic rates in MHC can be written as:^83^

[22] $$k_{ox/red}=\text{A}\int_{-\infty }^{\infty }\text{exp}\left(-\frac{{\left(\text{ξ}-\lambda \pm e\text{η}\right)}^{2}}{4\lambda k_{B}T}\right)\frac{d\text{ξ}}{1+\text{exp}\left(\text{ξ}/k_{B}T\right)},$$

where *A* is a pre-exponential factor that accounts for the electrode electronic density and strength, the plus corresponds to oxidation, the minus corresponds to redox, and ξ is the electron energy. The first integrated term of Eq. 22 represents the Marcus rate for electron transfer and the second integrated term accounts for the Femi-Dirac energy distribution.^83^ The molecular nature of the MHC provides more physical insight about the kinetics than the BV model; however, the MHC reaction rate model is given by a complex improper integral and is more computationally demanding. To reduce these complexities, the Faradic and exchange currents density for MHC can be fit to^83^

[23] $$i\approx \sqrt{\text{π}\lambda }\;\tanh\left(\frac{\text{η}}{2}\right)\text{erfc}\left[\frac{\lambda -\sqrt{1+\sqrt{\lambda }\;+{\text{η}}^{2}}}{2\sqrt{\lambda }}\right],$$

[24] $$i_{0}\approx \frac{\sqrt{\text{π}\lambda }}{2}\;\text{erfc}\left[\frac{\lambda -\sqrt{1+\sqrt{\lambda }\;}}{2\sqrt{\lambda }}\right],$$

Both Eq. 23 and Eq. 24 are valid over a wide range of λ, and the estimated error vanishes for large η and is less than 5% for small η.^83^

**Figure 5.**
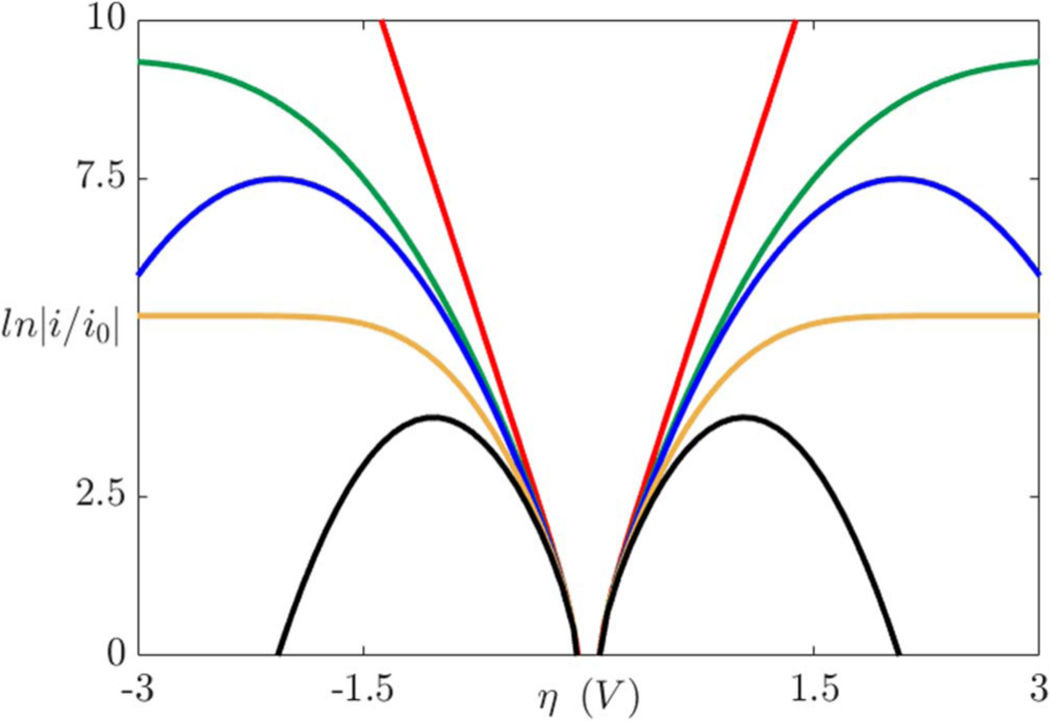
Variation of the current density as function of the overpotential: Butler-Volmer β = 0.5 (red line), Marcus-Hush-Chidsey λ = 30 (green line), Marcus λ = 30 (blue line), Marcus-Hush-Chidsey λ = 15 (yellow line), Marcus λ = 15 (black line). ^69^

To highlight the distinctive features between the BV, Marcus, and MHC models, the current density dependence on overpotential is presented in Figure 5.^69^ For small overpotentials, each of the models give approximately the same result. In contrast to the BV model where the current increases exponentially as a function of the overpotential, Marcus theory has a non-monotonic behavior as η increases in magnitude, which is not accurate. The MHC approach accurately captures both the saturation of the current and the monotonic dependence on the overpotential. This limiting behavior at high overpotentials exists due to the electronic density states of the electrode nature.^84^ Additionally, at these high overpotentials the BV model provides unrealistically large reaction kinetics.^83^ Finally, as λ increases, the current profiles of the BV model remain accurate for larger overpotential ranges.

## Bubble Detachment

**Figure 6.**
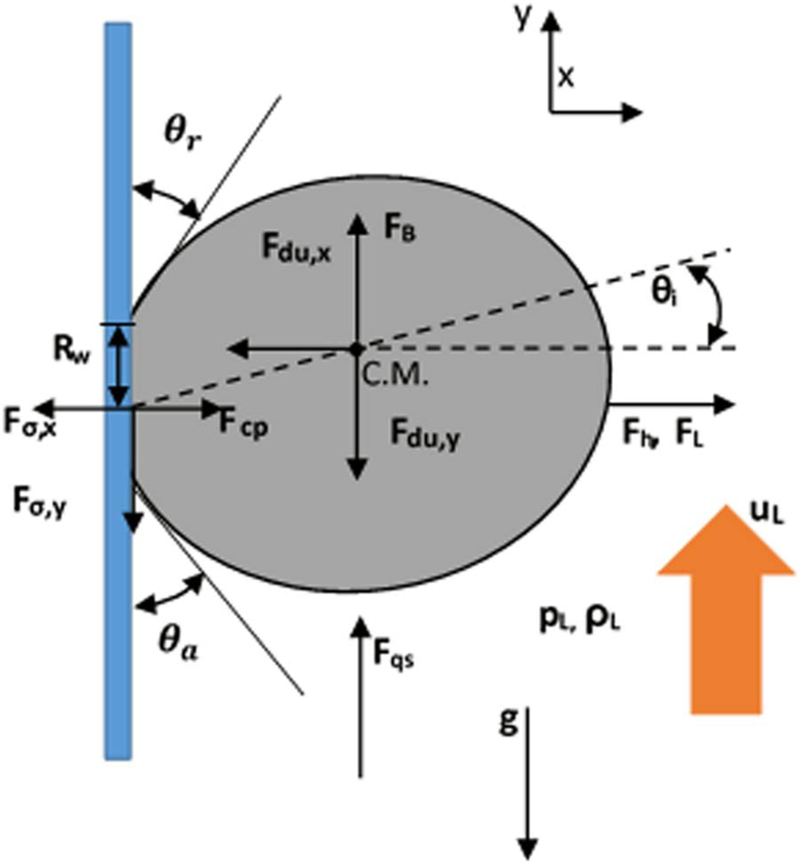
Hydrodynamics forces acting on a bubble at the electrode surface. The expressions of each force are defined in Table I. This figure is reproduced from Taqieddin et al., 2017.^2^

Bubbles continue to grow as long as there is a adsorption of dissolved gas, but as the bubble continues to get larger, the probability that it will detach from the electrode surface increases. This detachment process begins with the necking stage. At this stage, the bulk of the bubble begins to move away from the surface producing a region called the neck that continues to shrink until reaching zero thickness as shown in Figure 4. Once zero thickness is reached, the bubbles detach from the electrode surface and move toward the electrolyte bulk. The frequency of bubble detachment determines the efficiency of the gas-evolving electrodes since the detachment of bubbles uncovers the electrode surface allowing the bulk to react with the electrode surface.^85^ The detachment process can be treated as a quasi-static equilibrium process under the influence of several dynamic forces.^86^ Typically, these dynamic forces range from orders of 10^−8^ to 10^−6^ (N).^87,88^ Predicting bubble size at detachment can be done by balancing the applied dynamic forces at the bubble-liquid and bubble-electrode interfaces. The forces that act on a bubble at a general vertical solid surface during the detachment are summarized in Table I where the *x*-direction is normal to the solid surface and the *y*-direction is parallel as shown in Figure 6.^89,90^ Bubble detachment at a heat transfer surface has been extensively studied in which the bubble departure relative to a general wall is investigated.^89–92^ For gas-evolving electrochemical systems, bubbles at an electrode surface are further influenced by two additional forces: the electrostatic force, ***F**_e_*, and the Marangoni force ***F**_M_*.^86^

The electrostatic interactions between the electrode surface charge and the bubble surface can cause a premature departure of the bubble from the electrode surface, or ***F***_*e*_ may oppose the direction of bubble growth retaining the bubble at the electrode surface. The direction of ***F***_*e*_ depends on the surface chemistry of the electrode, bubble, bulk liquid interfaces, and the presence of adsorbing ions.^86^ The general expression, at the continuum scale, for a electrical force acting on a fluid is given as:^93,94^

[25] $$F_{e}=\int_{V_{\ell }}\left({\text{ρ}F}^{E}-\frac{\varepsilon_{0}E^{2}}{2}\nabla \varepsilon_{r}+\frac{\varepsilon_{0}}{2}\nabla \left(E^{2}\text{ρ}{\left(\frac{\partial \varepsilon_{r}}{\partial \text{ρ}}\right)}_{T}\right)\right)dV,$$

where *V*_ℓ_ (m^−3^) is the fluid volume, ρ_*F*_ (C m^−3^) is the free electric charge density, ***E*** (V m^−1^) is the electric field intensity, ε_*o*_ (8.85 × 10^−12^ C V^−1^ m^−1^) is the vacuum dielectric permittivity, $\varepsilon_{o}\;\varepsilon_{r}$ is the dimensionless, relative dielectric permittivity, and ρ is the fluid density. The first term in Eq. 25 models coulomb’s force when free charge builds up in the regime. The second term models the non-homogeneity of the dielectric permittivity. Finally, the last term accounts for the non-uniformity of ***E***, which induces pressure in the fluid.^94^ The contribution of ***F***_*e*_ can thus be repulsive or attractive based on which of these effects dominate the electrostatic interactions between the electrode and the bubble.

Marangoni stresses appear in the electrochemical systems due to the concentration gradients of the generated gases near the electrode surface.^86^ It is possible for the chemical reactions to produce surfactants, which may have concentrations that vary in space. It can be assumed that the concentration varies linearly within the Nernst diffusion layer, where the highest concentration occurs adjacent to the electrode. The thickness of this boundary layer decreases as the current density increases (i.e. the layer thickness varies from 10 to 100 μm for the current density range 10–100 mA cm^−2^.^6^ Concentration gradients of the released gas surfactant result in surface tension gradients that may significantly alter the bubble detachment frequency. ***F***_*M*_ can be expressed as^17^

[26] $$F_{M}=2\pi r^{2}\frac{\partial \text{σ}}{\partial \Gamma }\frac{\partial \Gamma }{\partial x},$$

where Γ (mol m^−2^) is the surfactant concentration and *x* is the direction normal to the electrode surface.^17^ The surfactant concentration gradient can be determined from on the diffusion boundary layer profile. Surface tension changes can be expressed as $\partial \text{σ}/\partial \Gamma =-\bar{R}T/(-\Gamma /\Gamma_{\infty })$, where Γ is the amount of adsorbed active surfactant through the bubble interface.^95^ The maximum packing concentration (surface excess of the solute), Γ_∞_, obeys Gibbs elasticity formula and can be modeled as

[27] $$\Gamma_{\infty }=\frac{-1}{\bar{R}T}\frac{d\text{σ}}{d\left(\ln a\right)},$$

where *a* is the solute activity, which accounts for the thermodynamic deviations from the ideal state of the chemical composition. Eq. 27 relates the overall interfacial affinity of the active species to the surface tension, which is determined by conducting experiments measuring the surface tension over a range of concentration.^96^ Changes in surface tension due to surfactant concentration have been experimentally measured.^97–99^

**Figure 7.**
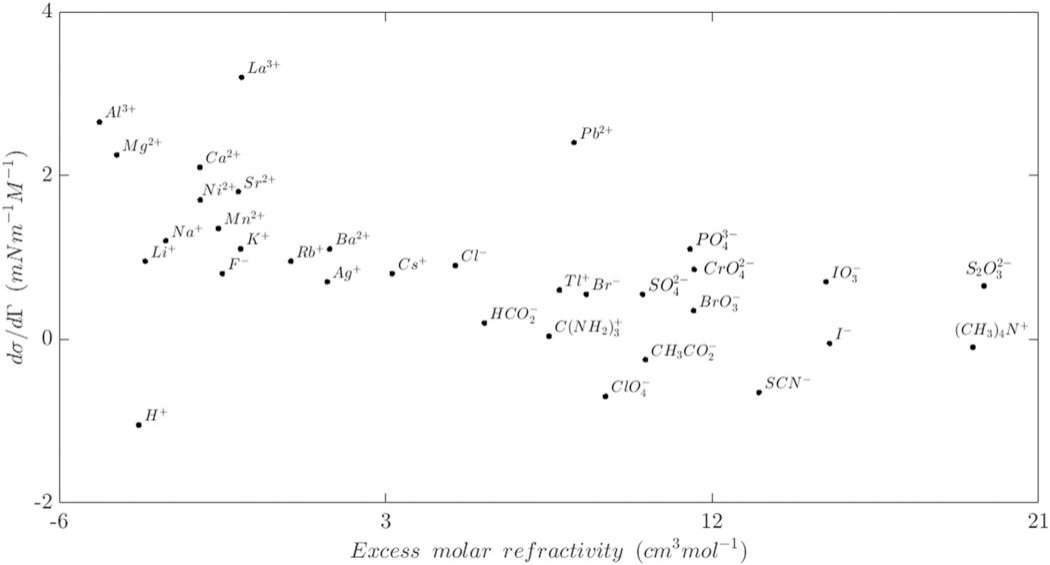
Ionic surface tension increments of aqueous ions at ambient conditions as a function of the excess molar refractivity.^98,99^

Modeling bubble detachment in gas-evolving electrochemical systems is complicated by the sensitive bubble dynamics on the electrode,^6^ the contact angle at the interfaces, and the geometry of the electrode surface.^86^ Bubbles may slide on the electrode surface if the sum of the acting parallel forces to the electrode surface is not zero. During this slipping movement, bubbles can coalesce to form a larger bubble, which can result in sudden detachment and return motion at the electrode.^6,9^ Bubble coalescence is currently an area of active research, so accurate models of the dynamics are developing. The sliding and coalescence at the electrode surface depend on the local velocity boundary condition, which are further complicated by the fact that the no-slip boundary condition is always valid.^100^ In fact, the hydrodynamic boundary conditions at the electrode surface depend on several operating conditions and physical parameters. There are three different slip types that are defined based on the physical interaction scales; slip at the scale of individual molecules, continuum slip at the liquid-solid boundary, and apparent slip due to the motion over heterogeneous boundaries such as bubbles. The strength of the slip condition at the electrode interface depends on the type and the quantity of the dissolved gases.^100^ In addition to the effects of pressure and temperature on the interfacial tension, the electrical properties of the electrolytic environment influence the surface tension behavior as well.^100^ The most important electrical property is the ionic strength of the aqueous solutions. Each ion has a unique surface tension sensitivity related to the ion activity.^97,99^ Figure 7 presents the value of ionic surface tension sensitivity.^98,99^

The contact angle as well as the advancing and receding angles also influence the along electrode bubble dynamics. Measuring the advancing and receding angles, θ_*a*_ and θ_*r*_ as shown in Figure 6, or considering the appropriateness of defining the contact angle as a macroscopic or microscopic quantity is difficult.^86^ The uncertainty of the contact angle complicates the modeling of the detachment forces. Geometric discontinuities of the electrode, such as porosity, are another source of bubble detachment. These surface features could prevent lateral growth, which leads to higher release frequency and variation in the bubble departure diameter.^86^ Once the bubble gets released from the electrode surface the rising stage of bubble’s life cycle begins.

Finally, the roughness of the electrode surface is one of the physical parameters that affects the surface tension values at the electrode surface. Both the roughness and the geometry of the electrode surface influence the dynamics of the nearby fluid, which can lead to changes in the adhesion forces that impacts the fluid-solid interactions at the electrode surface. As the roughness increases, the friction at the electrode surface increases lowering the opportunity for bubble slip to occur. However, the probability of slip can increase when there is a large accumulation of gas bubbles at the electrode solid boundary, especially at high roughness, which causes dewetting condition at the electrode surface. The wettability of the electrode surface is a function of the physicochemical properties of both solid and liquid states. To reduce bubble accumulation, surfactants are used to reduce the surface tension by using coating, sprays, or other chemical materials.^101^ Similarly, electrowetting techniques are used to enhance bubble detachment by modifying the wetting properties when an electric field is applied. This technique has physical effects on the interfacial contact angles.^102^

## Two-Phase Flow

Electrochemical gas-evolving systems are classified as two-phase flow system in which the bulk electrolyte is the liquid phase and the generated gas bubbles are the gas phase. These systems can be characterized as dispersed phase flow meaning that the gas bubbles are present throughout the continuous liquid phase and that the transfer of energy, momentum, and mass between the two phases has to be considered for individual bubbles.^103^ The main struggle in modeling dispersed phase flow is accurately capturing the exchanges between the two phases. The nonlinear and multiscale interphase interactions between the dissimilar phase particles result in a rich variety of flow regimes. Coupling and describing the interphase fluid physics can be done using different approaches based on the bubble-liquid interactions.

Generally, coupling the associated physics of the fluid flows can be done using either one-way, two-way or four-way coupling.^104,105^ One-way coupling is used in cases where the number of bubbles within the liquid is low (i.e. volume fraction is less than 10^−6^), where the liquid transfers momentum to the bubbles; however, the effect of the momentum from the bubbles to the liquid bulk is negligible. At higher volume fraction of the discrete phase (i.e. bubbles), the presence of bubbles can alter the liquid flow structure, so two-way coupling is needed when the value of the discrete phase volume fraction is between 10^−6^ to 10^−3^. Both one-way and two-way coupling can be implemented for dilute dispersed phase flow.^104^ In this particular flow, the dynamics of the bubbles are controlled by the fluid forces (drag and lift) and the flow can be estimated as collision free flow.^103^ The four-way coupling describes the dense dispersed phase flow where the collisions and continuous contact between the bubbles dominate.^103^ The dense flow takes place when the volume fraction of the discrete phase is higher than 10^−3^. Electrolytically generated bubble flow can be treated as dilute flow (i.e. volume fraction is less than 10^−3^) since the bubbles obey the fluid forces.^106–108^ The frequency of bubbles released from the electrode surface, the size of the generated bubbles, and the void fraction values play important role in reducing the collisions between the bubbles in the electrochemical cells. Thus, one and two-way coupling are the most frequently used models for electrochemical systems.

Modeling the flow interactions between the bubble and liquid depends on the resolution, where the fluid dynamics can be modeled using different equations depending on the time and length scales. Selecting the proper modeling scales depends on the fluid characteristics of the systems. At macro-scale modeling, the governing equations can be presented using the Euler, Navier-Stokes, or Burnett equations. At the smaller mesoscale, the molecular models of Boltzmann equations are used to model the flow fields using statistical and distribution functions. Micro-scale systems are modeled by tracking each individual fluid particle using Newton’s law, but this is generally only possible for rarefied gas systems. Given the need to model the electrolyte bulk at the scale of the electrochemical scale, the focus here is on the larger scale models.

Eulerian-Lagrangian (E-L) methodology precisely treats the dilute dispersed flow by expressing the liquid phase using the continuum model, and the gas bubbles as Lagrangian elements. In the E-L formulation, the bubbles are represented as dispersed particles and the electrolyte liquid as a carrier phase. The carrier or continuous phase is modeled in an Eulerian frame incorporating a statistical random field approach due to the averaging of the liquid fluctuations,^109^ and the particles or the bubbles are described in a Lagrangian frame as a stochastic point process.^110^ This process models the bubble with probability density functions to describe the bubbles as non-contiguous spheres of finite radius in the space. Flow fluctuations in the liquid phase are a consequence of the movement and momentum exchanged between the bubble and electrolyte. Even when the system is a laminar multiphase flow, it can experience significant fluctuations of pseudo-turbulent velocity with a range of spatial and temporal scales due to the bubbles.^110^ To model the influence of the bubble particles on the liquid and vice a versa, two-way coupling needs to be applied.

The dynamics of each individual bubble are modeled using Newton’s second law and the liquid phase model uses the Navier-Stokes equations. Interphase information such as mass transfer, momentum exchange, and energy transfer are continuously transfered between both the Lagrangian and the Eulerian phase.^111^ The E-L for-mulation is able to capture the nonlinear and multiscale interactions between the continuous and dispersed phases with high accuracy.^110^ By determining the instantaneous position, shape, and velocity of each individual bubble, the E-L approach is able to explicitly compute bubble-liquid and bubble-bubble collisions. Despite the advantages of the E-L approach, proper implementation of the interphase coupling is required to avoid numerical divergence.^110^ The E-L method only handles particles and continuum but a model of the interphase coupling with the property transfer at the surface is required to avoid numerical divergence. The following subsections present further details on the Eulerian and Lagrangian models as well as the dominant interphase and Multiphysics coupling models used for simulating two-phase flow in electrochemical systems.

### Eulerian phase and electrochemical processes—.

The liquid phase at the continuum scale models the mass and momentum transfers,assuming no energy transfer,using the incompressible continuity and Navier-Stokes equations as following:

[28] $$\frac{\partial \left({\text{ρ}}_{\ell }{\text{α}}_{\ell }\right)}{\partial t}+\nabla \cdot \left(\rho_{\ell }\alpha_{\ell }u_{\ell }\right)=-{\dot{M}}_{\ell \to b,}$$

[29] $$\frac{\partial \left({\text{ρ}}_{\ell }{\text{α}}_{\ell }u_{\ell }\right)}{\partial t}\;+\left({\text{ρ}}_{\ell }{\text{α}}_{\ell }u_{\ell }\cdot \nabla \right)=-{\text{α}}_{\ell }\nabla P+\nabla \cdot \left({\text{α}}_{\ell }{\text{τ}}_{\ell }\right)+\rho_{\ell }{\text{α}}_{\ell }g-f_{\ell \to b}+f_{e},$$

where ρ_ℓ_(kg m^−3^) is the electrolyte density, ${\text{α}}_{\ell }$ is the spatially varying liquid volume fraction [0,1], ***u***_ℓ_(m s^−1^) is the electrolyte velocity, ${\dot{M}}_{\ell \to b}$ (kg m^−3^ s^−1^) is the interphase mass transfer rate of dissolved gas in the liquid into and out of the gas bubbles, *P* is the pressure field, **τ**_ℓ_ (N m^−2^) is the shear stress tensor of the liquid phase, ***g*** (m s^−2^) is the gravitational constant, ***f***_ℓ → *b*_ (N m^−3^) is the volumetric forcing term due to liquid-bubble interactions that acts only at the interface, and ***f***_*e*_ (N m^−3^) is the acting electrical force on the fluid that comes from the ion species movement within the electrochemical system. The stress tensor for Newtonian and laminar flow, **τ**_ℓ_, in Eq. 29 is given by **τ**_ℓ_ = μ_ℓ_[(∇***u***_ℓ_) (∇***u***_ℓ_)^*T*^], where μ_ℓ_ (Pa s) is the molecular viscosity of the electrolyte. The momentum of the electrolyte and the bubble are linked in Eq. 29 by the volumetric momentum transfer term, ***f***_ℓ → *b*_. This term represents the sum of all instantaneous interfacial forces, over all bubbles, which are described in Table II as part of the Lagrangian phase formulation. The equations for the bubble are discussed in the Lagrangian phase section.

In Eq. 28, the total interphase mass transfer rate for the system, ${\dot{M}}_{\ell \to b}$, can be expressed as follows:

[30] $${\dot{M}}_{\ell \to b}=\frac{1}{V_{\ell }}\sum^{n}{\dot{m}}_{\ell \to b},$$

where *V*_ℓ_ (m^3^) is the liquid volume, *n* denotes the number of bubbles, and ${\dot{m}}_{\ell \to b}$ (kg s^−1^) is the mass transfer rate from the liquid to a bubble. The total amount of gas transfer through a particular bubble surface can be expressed as a summation over the chemical species as follows:

[31] $${\dot{M}}_{\ell \to b}=A_{b}\sum^{J}MW_{j}\dot{m}j^{\prime \prime },$$

where *J* is the number of species, *A*_*b*_ (m^2^) is the bubble surface area, *MW*_*j*_ (kg mol^−1^) is the molecular weight of the involved species, and ${\dot{m}}^{\prime \prime }$ (mol m^−2^ s^−1^) is the interfacial molar mass flux.

Due to the continuous motion and growth of gas bubbles at the electrode, the spatially and temporally varying value of α_ℓ_ changes during the electrochemical reactions. The change in α_ℓ_ values can also be associated with the transport of species since species concentration impacts the bubble nucleation, growth, and detachment at the electrode surface. The species transport near the electrode surface of a dilute solution can be modeled using the Nernst-Planck law:^73^

[32] $$N_{j}=c_{j}u_{\ell }-D_{j}\nabla c_{j}-\frac{z_{j}FD_{j}}{\bar{R}T}c_{j}\nabla U,$$

where ***N***_*j*_ is the species molar flux perpendicular to the electrode, *c*_*j*_ (mol m^−3^) is the concentration, *D*_*j*_ (m^2^ s^−1^) is the ionic diffusion coefficient of species *j*, *T* (K) is the species temperature, and *U* (V) denotes to the electric potential. Eq. 32 accounts for convection due to fluid flow, species diffusion, and electrically driven migration transport. While convection transport occurs in the bulk electrolyte, the diffusion takes place at the electrode surface between the viscous and double electric layers. Migration transport results from the motion of the charged species due to the electrical potential difference in the electrolyte.

The accumulated effect of the migration of charged species within the electrochemical system results in an electric current based.^13^ The value of the electric current density, *i* (A m^−2^), determines the production of the oxidized and reduced chemical species within the system,which can be given using Faraday’s law

[33] $$i=\sum^{J}z_{j}FN_{j}.$$

The current density direction is defined based on either being an oxidation or reduction current. In fact, ***i*** = ***i***_*ox*_ + ***i**_red_* where ***i***_*ox*_ is the oxidation current and ***i**_red_* is the reduction current. When ***i*** = 0, the chemical reactions are in equilibrium, which means the concentrations of the oxidation and reduction species remain constant and balanced. In this case, the equilibrium electrical potential, *U*_*eq*_ is determined as following:

[34] $$U_{eq}=U_{eq}^{o}+\frac{\bar{R}T}{nF}\text{ln}\left(\frac{c_{ox}}{c_{red}}\right),$$

where $U_{eq}^{o}$ is the standard electric potential tabulated for each oxidation and reduction reaction, *c*_*ox*_ is the concentration of the oxidization species at equilibrium, and *c*_*red*_ is the concentration of the reduction species at equilibrium. For non-equilibrium states, the concentration changes are described by the overpotential value which is given as:

[35] $$\text{η}\;\;\text{=}\;U-U_{eq}\;.$$

When η = 0, the value of ***i*** = 0 as shown in the electrochemical kinetics section of this paper. To apply an electrical potential to generate current density, the conservation of the current is

[36] ∇. i=0,

which indicates that the current does not accumulate locally in the electrolyte. The current conservation condition is required to calculate the electrolyte conductivity, which accounts for the presence of the gas bubbles within the electrolyte. To calculate the conductivity, Eq. 36 can be expressed in terms of Eq. 33 and Eq. 32, noting that the electroneutrality term ∑*z_j_c_j_* = 0 should be applied to ensure that there are no charged regions in the electrolyte at the macroscopic level.^13^

Gas bubbles inside the electrolyte are voids that affect the effective electrolyte conductivity *K* (S m^−1^) and the diffusion properties, *D_j_*. The effective electrolyte conductivity for the system is given as:

[37] $$K=\sum^{J}\frac{z_{j}^{2}F^{2}D_{j}}{\bar{R}T}c_{j}.$$

Aggregation of the gas bubble volume fraction, α_*b*_ = 1−α_ℓ_, decreases the value of *K*.^9^ Different equations have been reported to predict accurately *K* for heterogeneous media based on the homogeneous electrolyte conductance without gas bubbles as follows:^112–115^

**Figure 8.**
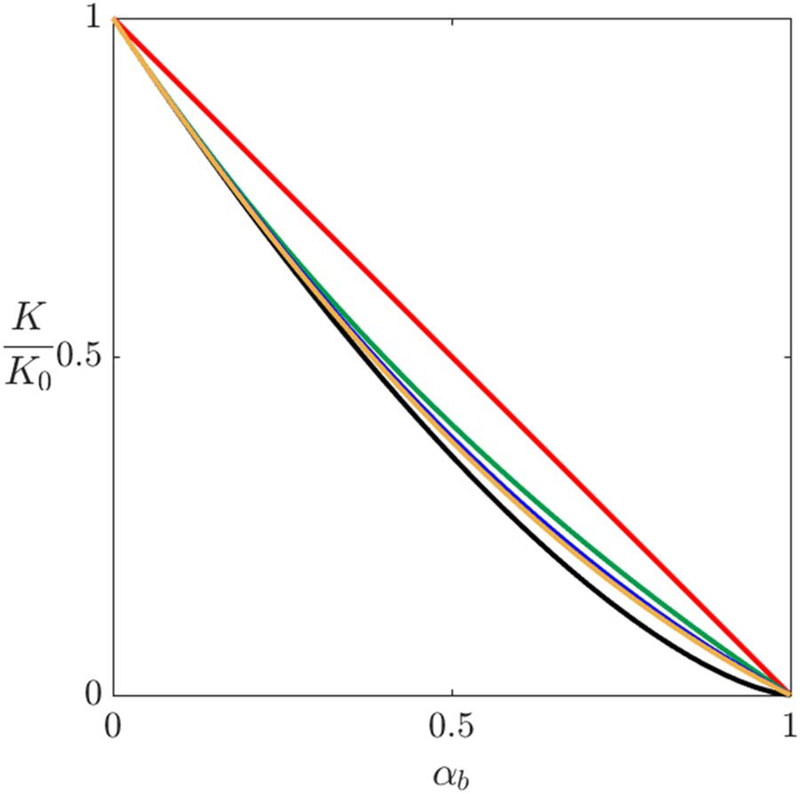
Reduction of electrolyte conductivity relations based on gas bubble volume fraction: Linear (red), Maxwell (green), Meredith/Tobias (blue), Prager (yellow), Bruggeman (black).

[38] $$\frac{K}{K_{0}}=\left\{ \begin{array}{l} \left(1-{\text{α}}_{b}\right)\;\;\;\;\;\;\;\;\;\;\;\;\;\;\;\;\;\;\;\;\;\;\;\;\;\;\;\;\;\;{\text{Linear}}^{\text{112}}\\ {\left(1-{\text{α}}_{b}\right)}^{1.5}\;\;\;\;\;\;\;\;\;\;\;\;\;\;\;\;\;\;\;\;\;\;\;\;\;\;\;{\text{Bruggeman}}^{113}\\ \left(1-{\text{α}}_{b}\right)/\left(1+0.5{\text{α}}_{b}\right)\;\;\;\;\;\;\;\;\;\;\;\;{\text{Maxwell}}^{\text{113}}\\ 8\left(2-{\text{α}}_{b}\right)\left(1-{\text{α}}_{b}\right)/\left(16-{\text{α}}_{b}^{2}\right)\;{\text{Meredith/Tobias}}^{\text{114}}\\ \left(1-1.5{\text{α}}_{b}+0.5{\text{α}}_{b}^{2}\right)\;\;\;\;\;\;\;\;\;\;\;\;\;\;\;{\text{Prager}}^{115} \end{array}\right.$$

where *K*_0_ is the electrolyte conductivity in the absence of gas bubbles. Figure 8 shows the conductivity ratios *K/K*_0_ as function of α_*b*_ based on the proposed relations in Eq. 38. The estimated value of *K* for α_*b*_ ≤0.3, is almost the same using any of the given relations in Eq. 38, excluding the linear relation. However, beyond that point, at which α_*b*_ ≥0.3, calculating K shows a variation up to 7% between the given models. For all cases, these functional dependencies are based on fits to experimental results. Other than the linear case, all of these models are similar; however the models by Bruggman, 1953^112^ and Maxwell, 1998^113^ are the ones predominantly used. The reduction of the effective conductivity due to bubble volume fraction, reflects the increase in overpotential since bubbles act as discontinuities that increases the electrical resistance. Similarly, the effective *D_i_* can be calculated using the relations in Eq. 38 to account for the presence of the gas bubbles.

### Lagrangian phase.—

Bubbles can be treated as rising particles that are best observed in the Lagrangian frame. The Lagrangian model describes the bubbles motion using Newton’s second law of motion, which connects the bubble kinematics to the acting force. In addition to the bubble dynamics, the interfacial mass transfer should be coupled to the model so that changes in the bubble volume can be accounted for. During the bubble motion, the acting forces on the bubble change the shape potentially resulting in changes to the mass transfer rates across the bubble. The Lagrangian model provides the ability to capture these effects with a higher accuracy than an exclusively Eulerian approach.^116^

In this microscopic frame, bubble compressibility can be neglected since the bubble volume does not change as a result of pressure or surface tension variations. There are extreme conditions where the bubbles can be compressible, but for standard electrochemical applications, these are not common.^117^ Instead, the volume of the bubble alters due to the gas diffusion across the bubble surface due to the electrochemical reactions. The density within the bubble can be assumed uniform and constant. The governing equations of an individual bubble’s trajectory and mass conservation is:

[39] $$\frac{d\left(x_{b}\right)}{dt}=u_{b},$$

[40] $$\frac{d\left(V_{b}\right)}{dt}=\frac{1}{{\text{ρ}}_{b}}{\dot{m}}_{\ell \to b}$$

where ***x***_*b*_ (m) is the position, ***u***_*b*_ is the velocity (m s^−1^), and *V_b_* is the volume (m^3^) of the bubble. The momentum conservation of each bubble can be expressed using Newton’s second law as follows:

[41] $$\rho_{b}V_{b}\frac{d(u_{b})}{dt}=F_{B}+F_{L}+F_{P}+F_{V.M}+F_{D}-{\text{ρ}}_{b}\frac{d({\text{V}}_{b})}{dt}u_{b},$$

where ***F***_*B*_, ***F***_*L*_, ***F***_*P*_, ***F***_*V.M*_, ***F***_*W*_, ***F***_*D*_ are respectively the buoyancy, lift, pressure, virtual mass, wall lubrication and drag forces (N).

The expression of each forcing term is presented in Table II. The dimensionless numbers that appear in Table II are *Re_b_* and *B_o_* which are the Reynold and Bond numbers, respectively. The numbers are defined as *Re_b_* = ρ_ℓ_|***u***_*b*_ − ***u***_ℓ_|*d_b_*/μ_ℓ_ and $Bo=(\rho_{b}-\rho_{\ell })gd_{b}^{2}/\sigma$, where *d_b_* (m) is the bubble diameter. The value *Re_b_* measures the ratio of the inertial force to the viscous force, and *Bo* measures the ratio of the gravitational force to the surface tension force. The characteristics of bubbles rising in the electrolyte can be determined by the values of *Re*_*b*_ and *Bo*.^122,123^ When surface tension forces dominate relative to gravitational, inertial, or viscous forces, the bubble resists surface deformations and keeps its spherical shape.^13^ When the forces are more balanced, the bubble can take on a variety of shapes.

**Figure 9.**
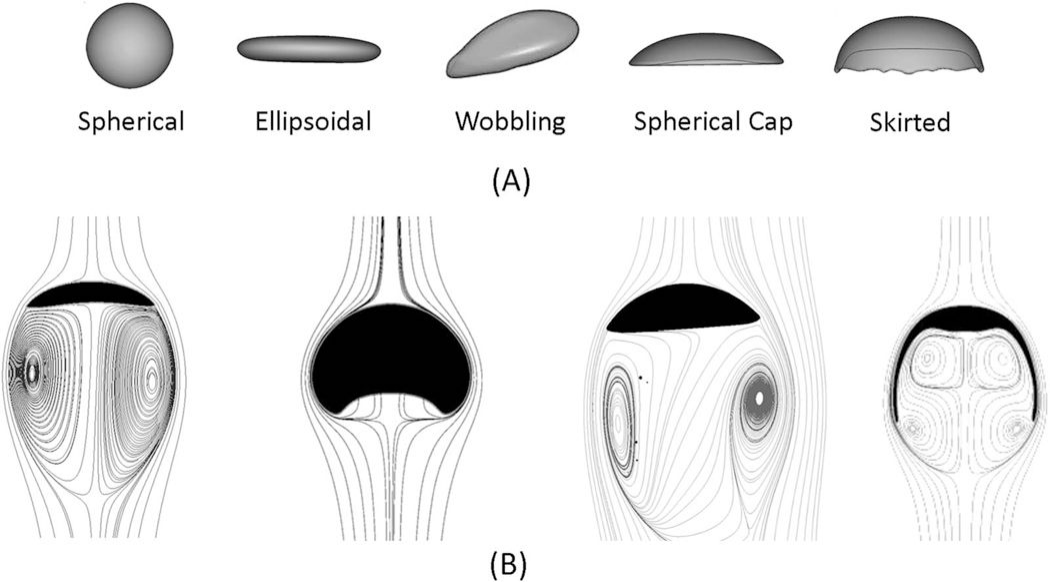
(A) Different shapes of a bubble rising in viscous liquid. The characteristic shape is set by the flow dynamics and fluid properties. (B) Instantaneous streamlines revealing the wakes associated with different bubbles. (From left to right) The rising spherical cap is characterized by a trailing ring wake. The spherical bubble has a smooth wake. Rising elliptical bubbles have a helical vortex wake, and skirted bubbles feature vortex ring wakes. The figure is reproduced from Gumulya et al., 2016.^126^

Generally, bubble shapes can be categorized into three main types: spherical/ellipsoidal, cap/skirted, and irregular/wobbling shapes.^123^ Figure 9 presents the different bubble shapes and the generated flow wakes based on the rising bubble in a stagnant liquid. The shape of the bubble and in particular the interfacial area, *A*_*b*_, has a significant influence on the overall mass transfer across the bubble surface. A bubble with a high interfacial area such as the skirted bubble will have a higher mass transfer rate across the bubble compared to a bubble with lower surface area such as spherical bubble. The mass transfer across the bubble interface changes the bubble volume which impacts the forces balance on the bubble and its rising velocity.^124,125^

Mass transfer rates between the bubble and liquid phases are controlled by the electrochemical reactions in the system which comes from either the difference in the electrical or chemical potentials between the bubbles and bulk solution.^127^ Determination of the mass transfer rates in electrochemical system requires evaluation of Nernst-Planck concentration fluxes in Eq. 32 and the Lagrangian mass transfer using Eq. 31. This mass transfer resulting from the electrochemical reactions, ${\dot{m}}_{\ell \to b}$, impacts both the mass conservation and force bal-ance as presented in Eq. 40 and Eq. 41, respectively. Further details of the interfacial mass transfer are provided in the next subsection.

### Interfacial mass transfer.—

Proper characterization of interfacial mass transfer requires a detailed consideration of the multiscale interactions around the bubble surface. While there are many factors that impact the rate of material transfer through the interface, there are three critical effects that must be accounted for in addition to accurately determining the bubble shape. First, the thermodynamic equilibrium state between the liquid-bubble phases impacts the phase change at the interface and is modeled using Henry’s law. Next, local velocity fluctuations due to turbulence can rapidly change the nearby concentration fields and enhance the mass transfer rates. Finally, the presence of contaminates inside the electrolyte changes the chemical mixture of the electrolyte, which varies the properties of the electrolyte such as the diffusion coefficient. These changes play a role in controlling the interfacial mass transfer.

Bubble geometry not only plays a role in calculating the interfacial area, but it also impacts the bubbles rising velocity. The rising velocity of the bubble depends on the nature of the electrochemical, physico-chemical system. The bulk liquid hydrodynamics and contamination physical properties, such as density and viscosity, affect the bubble motion by defining the viscous, surface tension, and buoyancy interactions between the two phases as discussed in the Lagrangian phase section. Several empirical and theoretical formulations have been developed to evaluate the terminal velocity of rising bubbles due to its importance in the multiphase flows.^122,128,129^ The bubble’s rising velocity influences the transport of species since the velocity determines the mean residence time of the bubble inside the liquid fluid. Also, the rising velocity of the ascending bubble determines the two-phase flow structure, which is characterized by bubble swarms, wakes and rising paths.

Qualitatively different bubble swarm and wake structures can be observed based on the value of Reynold number. At low Reynold numbers bubbles are expected to move in a straight path with a single-threaded wake. While at high Reynolds, the bubble experiences oscillatory motions, such as spiral and zigzag trajectories with a double-threaded wake.^130^ How the bubble rises also influences the structure of the wakes. Bubble wakes can be either steady without circulation, steady with circulation, or unsteady with vertex shedding. The induced wake eddies establish micro-convection that enhances the interfacial mass transfer rate at the bubble surface. These turbulent eddies can be suppressed in highly viscous systems. The shape of the bubble as it rises also plays a major role in the type of wake produced. As an example, the curvature of spherical bubbles is not conducive to generating a recirculating or vertex wakes. Elliptical or spherical-cap bubbles shed vorticity, which produces recirculating or eddy filled wakes that increases the interfacial mass transfer. The different types of wakes have qualitatively different mixing patterns and mass transfer boundary layer characteristics. Understanding the local mixing properties is valuable for chemical reaction boundaries, since the mixing has a considerable influence on the reaction yield and high selectivity.^131^

Although it is known that the vortex wakes produce very effective mass transport,^132^ the mass transfer from a bubble is very complex. The exchange of mass occurs within a diffusion layer in which the mass transfer is proportional to the concentration gradient at the bubble interface and is given in terms of Fick’s law.^133^ Table III shows a few correlations of mass transfer constants, *k*_*m*_, in terms of the dimensionless Sherwood number, *Sh* = *K*_*m*_ *L/D*_*i*_ where *L* is the characteristics length of the mass transfer. The value of *k*_*m*_ determines the mass transfer rate as shown in Eq. 8 where increases in *Sh* result in increases in the mass transfer rate. A high *Sh* indicates that convection dominates mass transfer compared to diffusive mass transfer, which generally occurs in high flow velocity such as turbulent flows. Another dimensionless number that impacts mass transfer rates is the Peclet number, *Pe*. Table III denotes the ratio of advection to diffusion mass transport parameters of flow velocity and diffusion coefficient, *Pe* = *uL/D*_*i*_.

## Recent Developments, Future Trends, and Challenges

Electrolytic gas-evolving systems have been investigated for more than a century.^139–141^ The great and continuous interest in developing the understating of electrolytic bubbles come from its wide and vital applications including hydrogen production,^142^ metal reduction cells,^143^ water electrolysis,^144^ and chlorine generation.^145^ In these applications, the presence of the gas bubbles is one of the dominant factors that controls the electrical efficiency of these systems and mass transport. To enhance bubble management in these systems, several techniques have been developed in the recent years to enhance bubble removal from the electrode surface and increase mass transfer mixing by either applying external fields such as super gravity,^146^ ultrasound,^147^ and magnetic-hydrodynamics^1^ fields. Alternatively, bubbles can be removed by means of manipulating the components of the electrochemical cell such as electrode shape,^148^ distance between the electrodes,^149^ and the cell/electrode inclination angle in the cell.^150^ However, these experimental investigations can be enhanced with numerical modeling to further enhance these techniques.

The majority of the experimental investigations have been limited to studying the nature of the micro-scale generated bubbles as reviewed by Sequeira et al., 2013,^9^ particularly, in studying the bubble growth and detachment at the electrode surface.^59,141,151^ Recently, the nature of nanoformed bubbles has received attention due to the difference in its fundamental behavior compared to the micro-bubbles.^152–154^ Nano-bubbles are considered more stable to dissolution against violent decompression and survive for longer compared to the micro-bubbles.^9,153^ Understanding the behavior of these bubbles is crucial to further enhance bubble management in electrochemical cells and the efficiency of these systems. Detecting these nano-bubbles and controlling them could impact industrial applications including nano-energy harvesting devices in which the bubbles are converted into electrical pulse.^155^ Developing models to describe properly the stability and life cycles of nano-bubbles is an important field of study^15^ as bubbles at this scale show deviation from the exiting models for example the contact angles of the nano-bubbles are overestimated by Young’s law.^9^

Another area of current investigation is the influence of the electrode structure on bubble development. Understanding bubble nucleation, growth, and removal from an electrode surface needs proper modeling that takes into account the electrode structure and complexity of the multi associated physics. In a recent investigation, Kady et al., 2016^85^ developed a structure-based optimization to describe the gas evolution at porous electrodes based on the structural properties of the electrode and the evolving bubbles by introducing preferential nucleation sites. This study shows the potential of further developing models to control bubble production for both hydrophobic and hydrophilic electrode structures. Deriving such models that incorpo-rates the characteristics of the electrode material will not only give better physicochemical insight of the bubble behavior, but it also provides opportunities to optimize the bubble-electrode interaction and, ultimately, develop new electrode materials and structures to manage these bubbles.

Significant progress in modeling of electrochemically gas-evolving bubbles has been presented, however, in most of these studies the modeling is focused on one or two stages of the bubble life cycle such as bubble formation,^156^ growth,^157^ or on simulating two-phase flows.^108^ However, full understanding of the nature of these bubble demands taking into account all the associated physics for the entire life cycle. This type of modeling helps to reveal several aspects in the area of gas-evolving systems including bubble nucleation at the electrode surface, effects of bubble presence on the electrolyte conductivity in large-scale systems, and voltage distribution on the gas-evolving electrodes.^9^ Multi-physics modeling also enables the simulation and study of complex new systems and applications where bubble evolution play an important role such as electrochemical therapy of tumors^158,159^ and nano-scale electrochemically energy harvesting and microfluidics devices.^155,160,161^ Performing a multiscale, multi-physics modeling to study electrolytic gas-evolving systems is a challenging task that requires a huge computational effort. To overcome the computational cost of multiscale multi-physics systems, optimizing the computational coupling between the multiscale physics is a topic that requires further attention.

## Conclusions

The life cycle of a bubble in electrochemically gas-evolving systems is defined by five stages cycle: nucleation, growth, detachment, rising, and burst of the bubble. Each stage is subject to multiscale and multiple physical phenomena influencing the bubble behavior. Further complicating the model is the electrochemical gas-evolving physics. The essential equations to describe the multiscale physics of gas bubbles in electrochemical applications are presented and reviewed. These phenomena range from quantum up to macroscopic scales. The wide range of physicochemical phenomena, in the electrolytic two-phase system, requires fundamental and detailed understanding of the relation between the different levels of the physical theories. Coupling these physical models can be achieved either through concurrent or sequential techniques. The concurrent approach is limited to the process of the smallest time step, and the sequential stitching model makes approximations to link different scale models into a unified model. A hybrid approach can enhance the coupling by bridging some processes through concurrent strategy and other processes using sequential method, but requires further development before application.

Understanding bubble nucleation at the electrode surface depends on the complex electrochemical processes. Nucleation rates in electrolytic medium are driven by the behavior of supersaturated dissolved gas in the electrolyte. There are two type of nucleation; homogeneous and heterogeneous nucleation. Presence of a substrate in heterogeneous nucleation lowers the nucleation activation energy compared to the homogeneous. The two prevailing nucleation theories, the classical nucleation theory and density functional theory, model nucleation from the macroscopic and molecular scale perspectives. The classical nucleation theory makes several assumptions to predict nucleation rates and is generally considered to underestimate the nucleation rate compared to experimental results. Since the density functional theory uses a microscopic scale, it has higher accuracy compared to classical nucleation theory; however, implementation of the density functional theory model to simulate bubble nucleation rates in electrolytic environment requires further investigations.

After nucleation, bubble growth takes place. The growth rate depends on the electrical current density magnitude, which drives the reaction rate and the development of regions of super saturated dissolved gas. Electrochemical kinetics play a significant role in evaluating the amount of produced gases and their local diffusion, where the amount of the produced gases controls the bubble nucleation, growth, and detachment of the bubbles at the electrode surface. The Butler-Volmer model evaluates the electrochemical kinetics from a mesoscopic viewpoint while Marcus theory determines the Faradic current density based on microscopic approach. Marcus-Hush-Chidsey model is a developed model of Marcus theory, which solves the electrochemical kinetics at a microscopic scale. Although the Butler-Volmer model gives similar results to Marcus-Hush-Chidsey model at small overpotential values, the BV calculations are overestimated at high overpotential values. Since the presence of gas bubbles in the electrolyte liquid phase increase the overpotential by reducing the effective conductivity of the bulk flow, evaluating the Faradic current density using Marcus-Hush-Chidsey approach is preferable in this case.

The bubble growth ends once the bubble detaches from the electrode surface. At the stage of bubble release, there are several hydrodynamic forces influencing the bubble’s quasi-equilibrium detachment, including the electrical field and the concentration fluctuations. Bubble response at the electrode surface is predominately determined by the interfacial conditions. These hydrodynamic conditions can be either slip or no-slip based on several parameters, such as the electrode roughness and geometric features. Electrode wettability is subject to physicochemical variations which is a function of the amount of dissolved gases, ionic strength of the electrolyte solution, electrode potential, and presence of chemical surfactant. These parameters determine the amount of interfacial slip occurrence, and therefore control bubble growth and detachment at the electrode surface.

Once the bubble detaches from the electrode surface it is free to move in the electrolyte resulting in a multiphase flow. To model the liquid electrolyte and the bubbles as particles, the Eulerian-Lagrangian approach provides the best balance of accurately and consistently incorporating the different physics. The Eulerian-Lagrangian model is a rigorous approach to describe the dispersed dilute flow of bubble rising and bursting. The two-phase computational framework of the model uses a statistical approach by modeling the bubble as a stochastic point process using a Lagrangian reference, while the liquid phase is described using a random field in an Eulerian frame. The dynamics of the multiphase flow are coupled with a concurrent mass transfer due to the transport of species between the two phases. Species transport result in ion flows within the electrolyte.

To accurately capture the interaction between the bubbles and the electrolyte it is necessarily to accurately model the interface location and the flux of properties and material through it. The interfacial mass transfer rates are a function of the liquid-gas interactions. The twophase interactions influence the shape, trajectory and wakes of the bubble. This fine scale modeling of the bubbles and their wakes can then be incorporated into system scale measurements of mass transfer. Modeling of the later quantities requires a careful mathematical approach, that accounts for all the physicochemical aspects of mass transfer at both macro- and micro-levels. Capture the interfacial mass transfer rates of electrolytic processes and at different scales is still an active area of research.

Accurately modeling the physicochemical behavior of bubbles in electrochemical cells is necessary to further enhance the overall efficiency of these cells. Modeling of the electrochemically gas-evolving cells requires a multiscale approach that unifies the multiphysics which happen during the proceeding chemical reactions. Separate, bubbles and electrochemical systems pose modeling challenges, but the combination of the two is the focus of a number of investigations. This review provides a summary of the fundamentals of these multiphysics by presenting the needed mathematical formulations to model an electrochemically gas-evolving system with bubbles.

While significant progress has been made in modeling bubbles and how they can be incorporated into models of electrochemical systems, there are a number of topics that are the focus of continued development. For example, accurate modeling of bubble nucleation in these systems would benefit from the application of the density functional theory approach. Also, coupling techniques of the multiscale physics in the gas-evolving is another area of possible development that will help to simulate these complex systems from the rest point, prior the chemical reactions take place, up to the bubble bursts in the system. Finally, continued development of the hybrid multiphysics schemes will enable greater resolution and more accurate simulations of these gas-evolving systems.

## Figures and Tables

**Table I.** Expression of interfacial forces acting on the bubble during growth at vertical electrode; *y*-axis is parallel to the electrode surface and *x*-axis is outward normal to the electrode surface.

Force (N)	Definition
*x*-direction forces	
$F_{s,x}=-{\text{d}}_{w}\sigma \frac{\pi }{{\text{θ}}_{a}-{\text{θ}}_{r}}\left(\cos{\text{θ}}_{a}-\cos{\text{θ}}_{r}\right)$	Surface tension force component; where d_w_ (m) is the bubble contact diameter, θ_*a*_ and θ_*r*_ are the advancing and receding contact angle
$F_{du,x}=-{\text{ρ}}_{l}\text{π}R^{\text{2}}\left(\frac{3}{2}C_{s}{\dot{R}}^{2}+R\ddot{R}\right)\;{\text{cosθ}}_{i}$	The unsteady growth force component, act as drag force; where C_*s*_ is a constant coefficient and θ_*i*_ is the inclination angle that is measured between the x-axis and the resultant force of the unsteady growth force
$F_{SL}=\frac{1}{2}C_{L}\rho_{\ell }{\text{π}R}^{\text{2}}{\left|u_{b}-u_{\ell }\right|}^{2}\text{ with }\;C_{L}=3.877{\text{γ}}^{1/2}{\left({Re}_{b}+0.014{\text{γ}}^{2}\right)}^{1/4}$	The shear lift force that acts; where *γ* is the dimensionless shear rate which can be determined by the turbulent flow profile for the liquid bulk phase
$F_{h}=\frac{9}{8}{\text{ρ}}_{\ell }{\left|u_{b}-u_{\ell }\right|}^{2}\frac{\text{π}d_{w}^{2}}{4}$	The hydrodynamic pressure force
$F_{cp}=\frac{2\sigma }{5R}\frac{\text{π}d_{w}^{2}}{4}$	The contact pressure force, due to the contact between the bubble curvature and the electrode solid surface
*y*-direction forces	
$F_{s,y}=-1.25d_{w}\sigma \frac{\text{π}\left({\text{θ}}_{a}-{\text{θ}}_{r}\right)}{\pi^{2}-{\left({\text{θ}}_{a}-{\text{θ}}_{r}\right)}^{2}}\left(\text{sin}{\text{θ}}_{a}-\text{sin}{\text{θ}}_{r}\right)$	Surface tension force component
$F_{du,y}\text{=}-{\text{ρ}}_{\ell }\text{π}R^{2}\left(\frac{3}{2}C_{s}{\dot{R}}^{2}+R\ddot{R}\right)\sin\;{\text{θ}}_{i}$	The unsteady growth force component
$F_{B}=\frac{4}{3}\text{π}R^{3}\left({\text{ρ}}_{l}-{\text{ρ}}_{b}\right)g$	The buoyancy force
$F_{qs}=6C_{D}{\text{μ}}_{\ell }\text{π}R\left|u_{\text{b}}-u_{\ell }\right|\text{ with }C_{D}=\frac{2}{3}\text{+}{\left[{\left(\frac{12}{Re_{b}}\right)}^{0.65}+0.796^{0.65}\right]}^{-1/0.65}$	The quasi-steady drag force, which acts in the flow direction

**Table II.** Expression of interfacial forces acting on the bubble in two-phase flow, the reference of each closure is cited in the reference column.

Force (N)	Closure	Reference
Buoyancy		
$F_{B}=\left({\text{ρ}}_{b}-{\text{ρ}}_{\ell }\right)V_{b}g$ Lift		
$F_{L}=-C_{L}{\text{ρ}}_{\ell }V_{b}(u_{b}-u_{\ell })\times (\nabla \times u_{\ell })$	$\begin{array}{l} C_{L}=\left\{ \begin{array}{l} \min\left\{0.288\text{tanh}(0.121r{\text{e}}_{b}),f(Bo_{d})\right\}\;\;Bo_{d}<4\\ f(Bo_{d})\;\;\;\;\;\;\;\;\;\;\;\;\;4<Bo_{d}<10\\ -0.27\;\;\;\;\;\;\;\;\;\;\;\;Bo_{d}>10 \end{array}\right.\\ f\left(Bo_{d}\right)=0.00105Bo_{d}^{3}-0.0159Bo_{d}^{2}-0.0204Bo_{d}+0.474\text{ and}\\ Bo_{d}=Bo{\left(1+0.163Bo^{0.757}\right)}^{2/3} \end{array}$	118
Pressure $F_{P}=-V_{b}\nabla P$ Virtual mass		
$F_{V.M}=-C_{V\mathrm{.M}}{\text{ρ}}_{\ell }V_{b}\left(\frac{Du_{b}}{Dt}-\frac{Du_{\ell }}{Dt}\right)$	$C_{V.M}=0.5$	119
$F_{W}=C_{W}V_{b}{\text{ρ}}_{\ell }{\left|u_{b}-u_{\ell }\right|}^{2}n_{W}$	$\begin{array}{l} C_{W}=C_{WLr}^{b}\left(\frac{1}{y_{W}^{2}}-\frac{1}{{\left(d_{cell}-yW\right)}^{2}}\right)\\ n_{W}:\text{wall normal vector}\\ C_{WL}=\left\{ \begin{array}{l} 0.47\;\;\;\;\;\;\;\;\;Bo<1\\ \text{exp}\left(-0.933B_{o}+0.179\right)\;1<\;Bo\;<\;5\\ 0.00599B_{o}-0.0187\;\;\;5<Bo<33\\ 0.179\;\;\;\;\;\;\;\;Bo>33 \end{array}\right.\\ d_{cell}:\text{electrochemical}\;\text{cell/reactor}\;\text{diameter(m)}\\ y_{W}:t\text{he}\;\text{near wall distance}\;\text{(m)} \end{array}$	120
Drag $F_{D}=-\frac{1}{8}C_{D}{\text{ρ}}_{\ell }\pi d_{b}^{2}\left|u_{b}-u_{\ell }\right|(u_{b}-u_{\ell })$	CD=max{min{1Reb(1+0.15Reb0.687),48Reb},83BoBo+4}	121

**Table III.** Expression of interfacial mass transfer constant correlations in terms of Sherwood number, *Sh*, as reported by literature.

Relation	Physical conditions
$Sh=1+{\left(1+0.564Pe^{2/3}\right)}^{3/4}$	Valid for creeping flow^123^
$Sh=\frac{2}{\sqrt{\pi }}{\left[1-\frac{2.89}{R{\text{e}}_{b}^{1/2}}\right]}^{1/2}Pe^{1/2}$	*Re_b_* > 70^123^
$Sh=\frac{2}{\sqrt{\pi }}Pe^{1/2}$	Assuming very thin concentration layer and potential flow around the bubble^134^
$Sh=\frac{2}{\sqrt{\pi }}{\left[1-\frac{2}{3}\frac{2.89}{{\left(1+0.09R{\text{e}}_{b}^{2/3}\right)}^{3/4}}\right]}^{1/2}\left(2.5+Pe^{1/2}\right)$	*Re*_*b*_ < 100 *and P*_*e*_ > 1^135^
$Sh=\frac{2}{\sqrt{\pi }}Pe^{1/2}AR\left(\text{χ}\right)\;\text{where }AR\left(\text{χ}\right)=0.524+0.88\text{χ}-0.49{\text{χ}}^{2}+0.086{\text{χ}}^{3}$	Valid for bubble rising in a stationary flow, where χ is the aspect ratio of the deformed bubble shape. The relation is valid over the following ranges: 500 *< Re*_*b*_ *<* 100, *P*_*e*_ > 100*Re*_*b*_ and 1 < χ < 3^136^
$Sh=1.13{\left[1-\frac{2+3\mu_{\text{b}}/\mu \text{l}}{1+{\left(\rho_{\text{b}}\mu_{b}/\rho \text{l}\mu \text{l}\right)}^{1/2}}\frac{1.45}{\text{R}{\text{e}}_{b}}\right]}^{1/2}Pe^{1/2}$	Valid only when internal circulation is complete, flow separation is negligible, and high *Re_b_*^137^
$Sh=1+{\left[1+{\left(\frac{4}{3\pi }\right)}^{2/3}{\left(2Pe_{\max}\right)}^{2/3}\right]}^{3/4}$	*Pemax* is evaluated based on the maximum velocity of the liquid phase at the interface. The relation is valid over the following ranges: 0.1 < *Re_b_* < 100 and 1 < *Pe* < 2000^138^
